# The m^5^C methyltransferase NSUN2 promotes codon‐dependent oncogenic translation by stabilising tRNA in anaplastic thyroid cancer

**DOI:** 10.1002/ctm2.1466

**Published:** 2023-11-20

**Authors:** Peng Li, Wenlong Wang, Ruixin Zhou, Ying Ding, Xinying Li

**Affiliations:** ^1^ Department of General Surgery Xiangya Hospital Central South University Changsha Hunan China; ^2^ National Clinical Research Center for Geriatric Disorders Xiangya Hospital Central South University Changsha Hunan Province China; ^3^ Department of Hepatobiliary Surgery Sichuan Provincial People's Hospital School of Medicine University of Electronic Science and Technology of China Chengdu China

**Keywords:** anaplastic thyroid cancer, c‐Myc, codon, drug‐resistance, global translation, leucine, m^5^C, NSUN2, tRNA

## Abstract

**Background:**

Translation dysregulation plays a crucial role in tumourigenesis and cancer progression. Oncogenic translation relies on the stability and availability of tRNAs for protein synthesis, making them potential targets for cancer therapy.

**Methods:**

This study performed immunohistochemistry analysis to assess NSUN2 levels in thyroid cancer. Furthermore, to elucidate the impact of NSUN2 on anaplastic thyroid cancer (ATC) malignancy, phenotypic assays were conducted. Drug inhibition and time‐dependent plots were employed to analyse drug resistance. Liquid chromatography–mass spectrometry and bisulphite sequencing were used to investigate the m^5^C methylation of tRNA at both global and single‐base levels. Puromycin intake and high‐frequency codon reporter assays verified the protein translation level. By combining mRNA and ribosome profiling, a series of downstream proteins and codon usage bias were identified. The acquired data were further validated by tRNA sequencing.

**Results:**

This study observed that the tRNA m^5^C methyltransferase NSUN2 was up‐regulated in ATC and is associated with dedifferentiation. Furthermore, NSUN2 knockdown repressed ATC formation, proliferation, invasion and migration both in vivo and in vitro. Moreover, NSUN2 repression enhanced the sensitivity of ATC to genotoxic drugs. Mechanically, NSUN2 catalyses tRNA structure‐related m^5^C modification, stabilising tRNA that maintains homeostasis and rapidly transports amino acids, particularly leucine. This stable tRNA has a substantially increased efficiency necessary to support a pro‐cancer translation program including c‐Myc, BCL2, RAB31, JUNB and TRAF2. Additionally, the NSUN2‐mediated variations in m5C levels and different tRNA Leu iso‐decoder families, partially contribute to a codon‐dependent translation bias. Surprisingly, targeting NSUN2 disrupted the c‐Myc to NSUN2 cycle in ATC.

**Conclusions:**

This research revealed that a pro‐tumour m5C methyltransferase, dynamic tRNA stability regulation and downstream oncogenes, c‐Myc, elicits a codon‐dependent oncogenic translation network that enhances ATC growth and formation. Furthermore, it provides new opportunities for targeting translation reprogramming in cancer cells.

## Graphical abstract

In brief

P. Li et al. reports the m^5^C methyltransferase NSUN2 catalyzes tRNA m^5^C modification and regulates tRNA stability that is required for oncoprotein translation reprogramming and vicious dedifferentiation in anaplastic thyroid cancer cells. The study provides new opportunities for targetingcodon‐dependent oncogenic translation network.

1. NSUN2 extensively catalyzes the tRNA m^5^C modification, which is associated with tRNA secondary structure.

2. NSUN2 regulates tRNA stability more than aminoacylation to support selectivecodon‐dependent oncoprotein synthesis.

3. NSUN2 promotes maturation of its upstream transcription factor, c‐Myc.

4. Targeting NSUN2 exhibited broad anti‐cancer effects in vitro and in vivo.

5. NSUN2 maintains drug resistance in cancer cells.

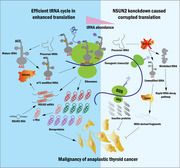

## 1 INTRODUCTION

Thyroid cancer is the most common endocrine malignancy.^1^ Differentiated thyroid cancer (DTC), including follicular thyroid cancer and papillary thyroid cancer (PTC), account for up to 85−90% of all thyroid cancer cases. DTC patients usually indicate a favourable prognosis after receiving standardised treatment.^2^, ^3^ Whereas anaplastic thyroid cancer (ATC) is a rare but clinically fatal malignancy characterised by dedifferentiation and clonal expansion of immature cells.^4^, ^5^ Currently, ATC treatment remains ineffective for prolonging overall survival because of its high metastasis rate and resistance to most therapies, including radio‐iodine ablation, chemotherapy and external‐beam radiotherapy. Therefore, elucidating the molecular mechanisms underlying ATC initiation and progression and identifying novel candidate targets to improve therapeutic strategies is of great significance.

Significant proteomic expression reprogramming is characterised by enhanced global translation in tumourigenesis and progression and has become a primary focus in cancer research.^6^ Translation is mediated by charged transfer RNAs (tRNAs), ribosomes and chaperone proteins, which decode genomic transcripts. Cellular tRNA is the most extensively modified RNA,^7^ and its modifications regulate its stability and anticodon–codon interactions on the ribosome, ensuring efficient and accurate protein synthesis.^8^, ^9^, ^10^ Dysregulated tRNA modifications are widely observed in human diseases, such as cancer,^11^ metabolic disorders^12^, ^13^ and intellectual disability.^14^, ^15^

One of the most abundant tRNA modifications is 5‐methylcytosine (m^5^C). It is often clustered in the cytosine at the junction region between the T‐stem and variable loop.^8^, ^16^, ^17^, ^18^ Catalysation of m^5^C modification involves NSUN2 (NOP2/Sun domain family member 2) and Myc‐Induced SUN‐Domain‐Containing Protein (Misu) methyltransferases, and they have been linked to numerous pathological and physiological processes, including cell proliferation,^19^ stress response^20^ and exosomes.^21^ Previous studies^22^, ^23^ revealed that NSUN2 introduces 5‐methylcytidines into cytoplasmic and mitochondrial tRNAs, whereas its knockdown profoundly inhibits translation.^24^, ^25^ However, the oncogenic functions and the precise underlying mechanisms of tRNA m^5^C modifications in cancers, specifically in ATC, remain unknown.

This study demonstrates that NSUN2 is significantly up‐regulated in ATC and is associated with poor prognosis. Impaired tRNA m^5^C modification upon NSUN2 protein knockdown inhibits ATC initiation and progression in vivo and in vitro. Mechanically, NSUN2‐mediated tRNA m^5^C modification promotes the translation of a biased transcript subset, including proto‐oncogenic transcription factors and anti‐apoptosis proteins, using the m^5^C‐modified stable tRNA‐decoded codon. Clinically, NSUN2 knockdown enhances the chemosensitivity of ATC cells. Collectively, the data acquired from this study reveal a significant link between NSUN2‐mediated m^5^C tRNA modification and ATC progression, providing a novel molecular basis for developing potential therapeutic strategies against ATC.

## 2 RESULTS

### 2.1 NSUN2 is up‐regulated in ATC

**FIGURE 1:**
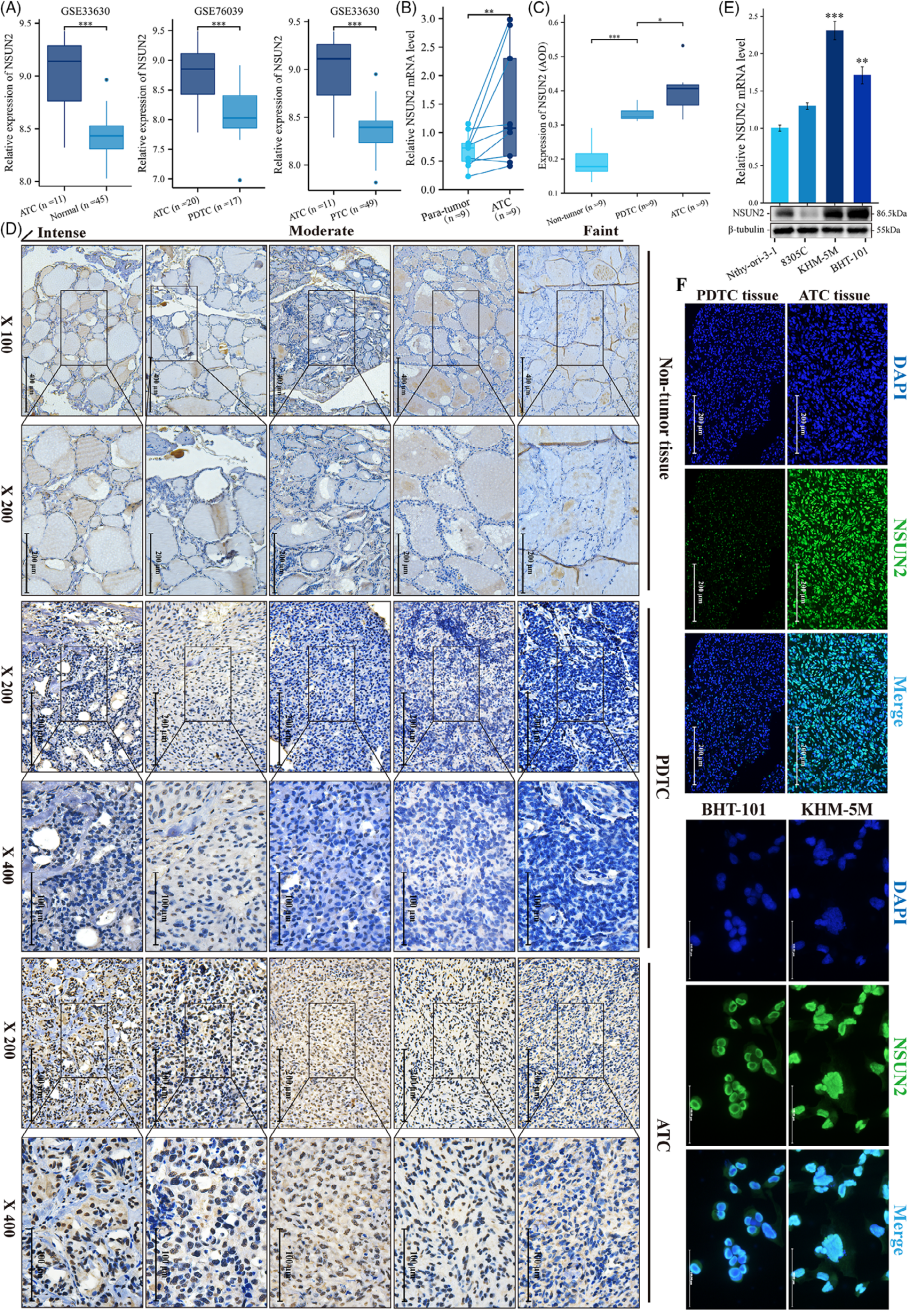
Expression of NSUN2 in anaplastic thyroid cancer. (A) Box plots showing the expression of NSUN2 between ATC and normal, PDTC or PTC samples from two different publicly available RNA‐seq datasets GSE33630 and GSE76039. (B) qRT‐PCR showing the relative expression of NSUN2 in ATC (*n* = 9) and paired para‐tumour tissue (*n* = 9). Wilcoxon signed rank test, *p* = 0.008. (C) Quantification of NSUN2 staining in normal thyroid, PDTC and ATC tissue by average optical density (AOD). Tukey HSD test. (D) IHC staining for NSUN2 in normal thyroid, PDTC and ATC tissue. Grouped by faint, moderate and intense staining. (E) qRT‐PCR and WB showing the relative expression of NSUN2 in ATC and normal thyroid cell lines. Wilcoxon signed rank test. (F) IF staining showed NSUN2 (green fluorescence) located in nuclear.

To explore the m^5^C modification system in thyroid cancer, a comprehensive analysis was conducted via RNA expression data from four independent accessible thyroid cancer studies (accession GSE76039,^26^ GSE33630,^27^ GSE65144^28^ and GSE29265). It was revealed that NSUN2 mRNA was markedly elevated in ATC compared with PTC, poorly differentiated thyroid cancer (PDTC), or normal thyroid tissue (Figures 1A and S1A and D). Furthermore, the OncoPrint map of the NSUN2 gene of thyroid cancer patients in Memorial Sloan Kettering Cancer Center and The Cancer Genome Atlas (TCGA) datasets indicated <0.2% genetic alteration (Figure S1B). Subsequently, thyroid cancer samples from the TCGA dataset were categorised into two groups based on their NSUN2 level. Substantially high NSUN2 expression was associated with significantly worse overall survival of PTC patients in TCGA (Figure S1C). The pathway analysis identified several enriched pathways in the NSUN2^high^ group than the NSUN2^low^ group, including proteolysis, RNA metabolism, cellular response to DNA damage stimulus, cell cycle and signalling by Rho GTPases (Figure S1E). These findings suggest a potential regulatory role of NSUN2 in key cellular processes and its association with the aggressiveness of thyroid cancer.

The expression of NSUN2 in thyroid cancer patients was also elucidated to validate the aforementioned results. Consistent with previous results, the acquired data revealed that NSUN2 expression was elevated in ATC samples compared to matched para‐tumour tissues (Figure 1B). Additionally, immunofluorescence (IF) and immunohistochemistry (IHC) staining indicated a specific up‐regulation of NSUN2 in ATC than in poorly DTC (Figures 1C, D and F). Moreover, NSUN2 was also up‐regulated in ATC cell lines, KHM‐5 M and BHT‐101 (Figures 1E and F). Overall, these data revealed that NSUN2 was significantly up‐regulated in ATCs and was also associated with poorly differentiated status.

### 2.2 NSUN2 promotes ATC cancer progression

**FIGURE 2:**
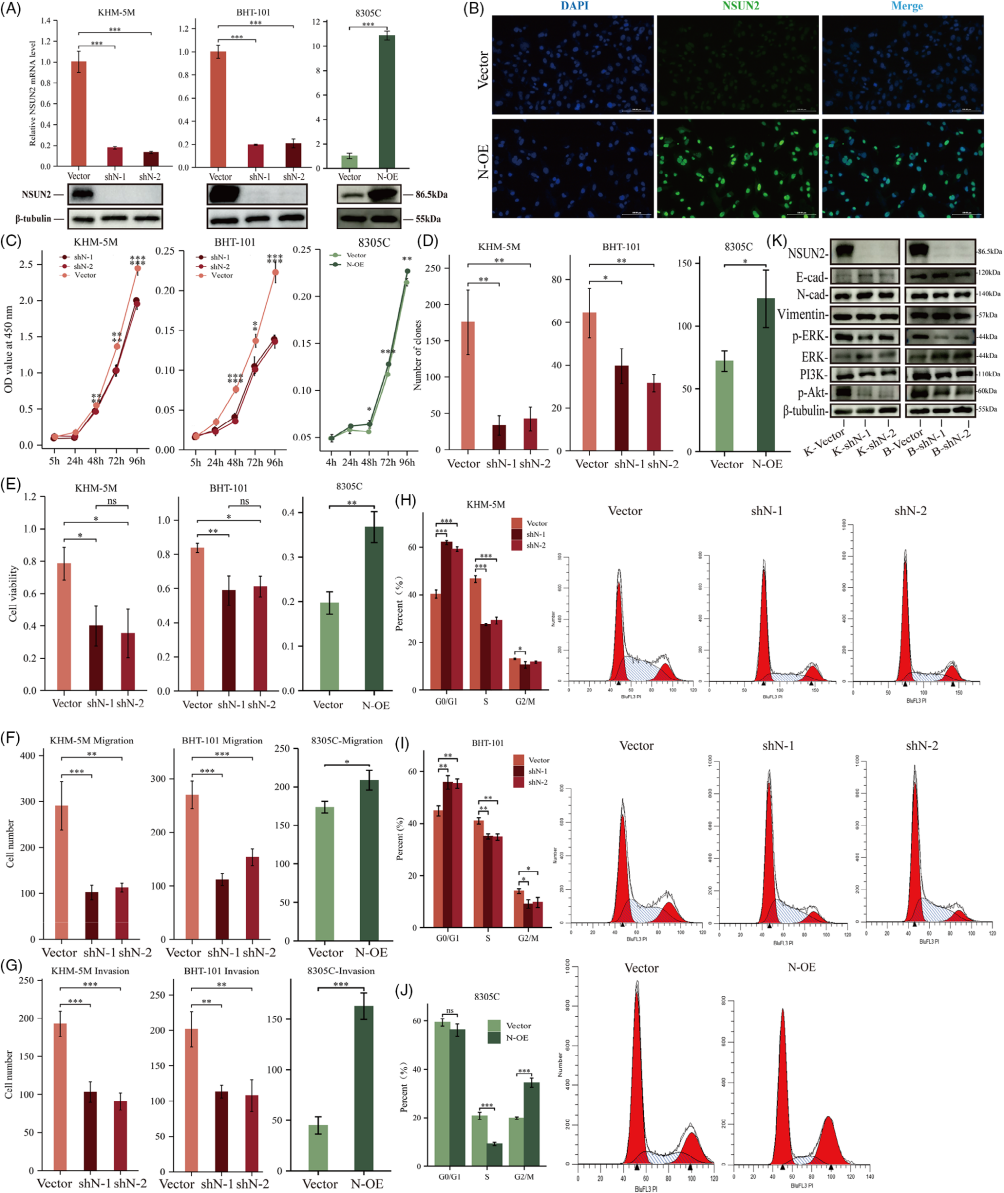
NSUN2 promotes malignancy of ATC. (A) qPCR and WB validated the expression level of NSUN2 in vector, shN and N‐OE cell lines. Tukey HSD test. (B) IF staining showed overexpressed NSUN2 (green fluorescence) located in nuclear. (C) CCK‐8 assay screening for effects of NSUN2 knockdown on KHM‐5 M and BHT‐101, NSUN2 overexpression on 8305C proliferation from day 0 to day 4. Pairwise comparisons of estimated marginal means. (D) Statistical analysis of colony formation for vector, shN and N‐OE. (E) Statistical analysis of EdU assay for vector, shN and N‐OE. (F and G) Quantitative analysis of vector, shN and N‐OE cell migration (F) and invasion (G) assessed by in vitro transwell assay. (H–J) Flow cytometry showed NSUN2 promotes ATC cells transition from G0/G1 phase to S phase and G2/M phase. (K) WB shows changes of key protein expression in representative signal pathways in vector and shN cell.

By using two short hairpin (shN‐1 and shN‐2) and a self‐amplifying (N‐OE) RNA, NSUN2 was knocked down in KHM‐5 M and BHT‐101 ATC cells and overexpressed in 8305C, respectively (Figures 2A and B). Dynamic (Figure 2C), static (Figures 2E and S2B) and macroscopic (Figures 2D and S2D) level evidence showed knockdown of NSUN2 significantly inhibited ATC cell proliferation. Additionally, NSUN2 depletion also impaired the invasion and migration abilities of both KHM‐5 M and BHT‐101 cells (Figures 2F, G and S2C). However, NSUN2 overexpression endowed 8305C cells with increased proliferation, colony formation, invasion and migration capabilities (Figures 2C–G and S2E–G). Moreover, flow cytometry analysis revealed that NSUN2‐depletion caused cell cycle arrest in ATC cells (Figures 2H and I), whereas its overexpression promoted the progression of the cell cycle in 8305C (Figure 2J). Additionally, the influence of NSUN2 on different key pathways was assessed, which revealed that NSUN2 inhibition down‐regulated the expression of p‐ERK, p‐Akt and PI3K in ATC cells (Figure 2K and S3D). Taken together, these data revealed various essential functions of NSUN2 in ATC progression in vitro.

### 2.3 Inhibition of NSUN2 in ATC reduces tolerance to chemotherapy drugs

**FIGURE 3:**
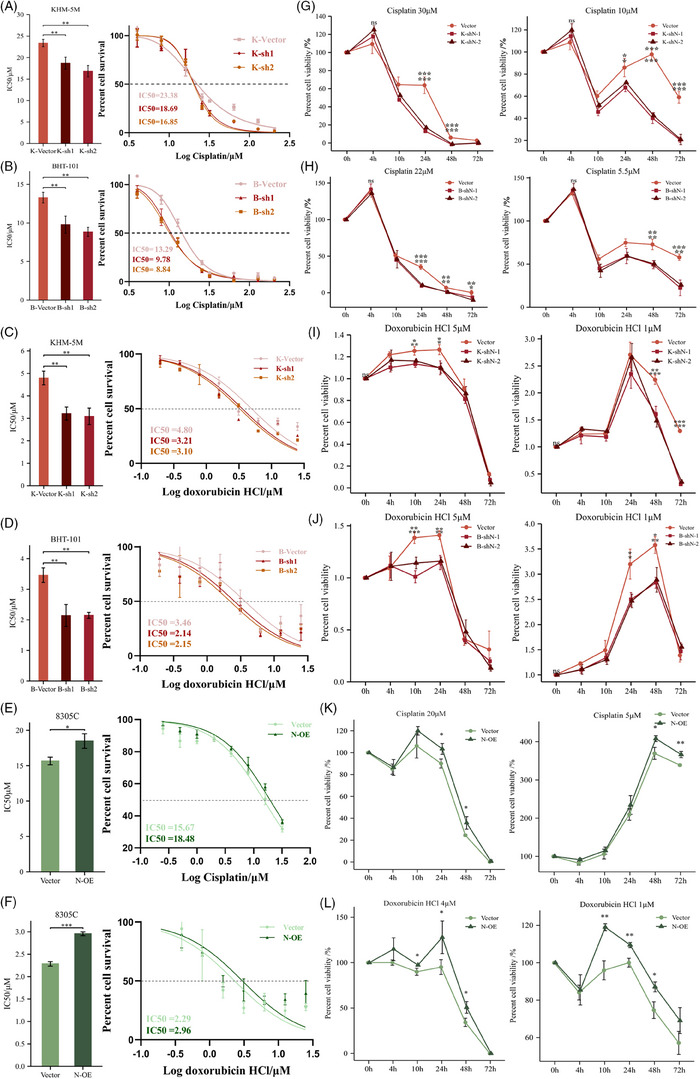
IC50 and time‐dependent curves of ATC cells exposed to cisplatin and doxorubicin HCl. (A–F) Cell growth inhibitory assay and statistical comparisons to evaluate the impacts of NSUN2 knockdown on the response of KHM‐5 M and BHT‐101 cells to cisplatin and doxorubicin HCl (A–D). Cell growth inhibitory assay and statistical comparisons to evaluate the impacts of NSUN2 overexpression on the response of 8305C cells to cisplatin (E) and doxorubicin HCl (F). IC50: half‐maximal inhibitory concentration. Tukey HSD test. (G–L) Time plot of the response of vector, shN and N‐OE to high or low concentrations of cisplatin and doxorubicin HCl. Pairwise comparisons of estimated marginal means.

NSun2−/− cells in skin tumours are highly sensitive to anti‐cancer drugs.^23^ Here, it was revealed that the cellular response to the DNA damage stimulus pathway was enriched in the NSUN2^high^ group, as indicated previously in Figure S1E. Inferring that NSUN2 also plays an important role in the tolerance of ATC to chemotherapy drugs. To investigate this hypothesis, the half‐maximal inhibitory concentration (IC_50_) of doxorubicin HCl and cisplatin was determined in cancer cells with NSUN2 knockdown or overexpression. NSUN2 knockdown in KHM‐5 M and BHT‐101 cells indicated significantly decreased IC_50_ values (Figures 3A–D and Table S1), whereas its overexpression in 8305C revealed increased IC_50_ values of doxorubicin HCl and cisplatin (Figures 3E and F and Table S1). Moreover, the time‐dependent plots further showed that NSUN2 knockdown shortened the plateau phase and improved the efficacy of chemotherapy drugs (Figures 3G–J). NSUN2 knockdown decreased the tolerance of ATC cells cisplatin or doxorubicin HCl under low‐dose treatment (Figures 3G–J). Whereas the survival of NSUN2‐overexpressed 8305C increased significantly when exposed to both chemotherapy drugs (Figures 3K and L). These results demonstrated that NSUN2 could facilitate the acquisition of drug resistance in 8305C cells. Thus, conventional chemotherapeutic agents combined with RNA cytosine‐5 methylation inhibitors may provide an effective anti‐ATC strategy.

### 2.4 Targeting NSUN2 expression inhibits ATC progression and drug resistance in vivo

**FIGURE 4:**
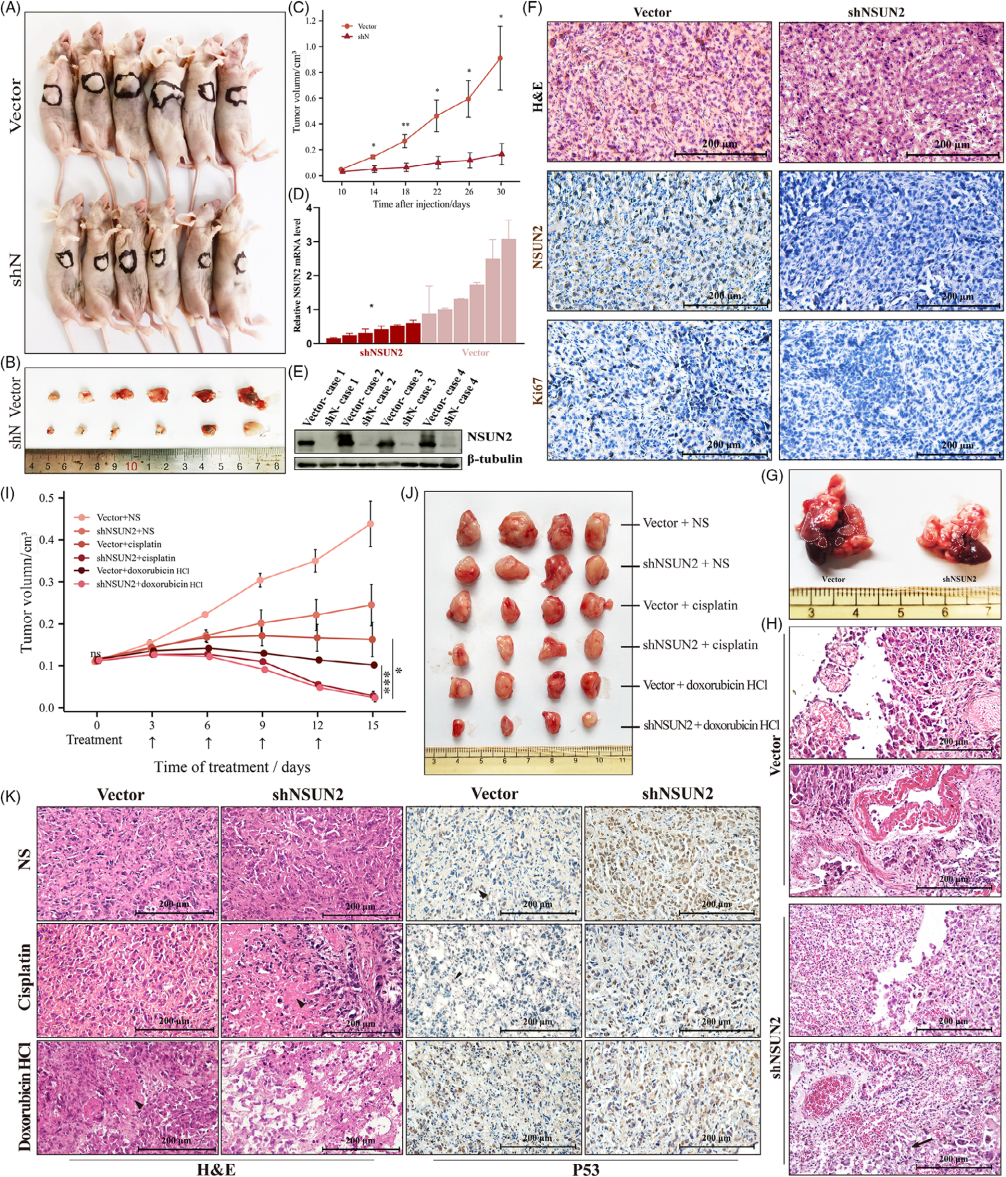
NSUN2 knockdown slows ATC growth in vivo and lung metastasis, increasing the efficacy of chemotherapy drugs. (A and B) Overview of tumours in xenograft mice model subcutaneously implanted with shN and vector cells. Scale bars, 200 μm. (C) Growth curves of tumour volumes formed by shN and vector cells. Data were presented as the mean ± SEM. (D) qRT‐PCR analysis of NSUN2 mRNA level in xenograft tumours. (E) WB showing the protein level of NSUN2 in xenograft tumours. (F) Haematoxylin–eosin staining and IHC staining of NSUN2 and Ki67 in tumour samples in xenograft mice model subcutaneously implanted with shN and vector cells. Scale bars, 200 μm. (G) Representative images of tumours in lung metastasis mice model intracardiac‐injected with shN and vector cells. shN cells injection developed less metastatic nodules than vector cells injection. (H) Haematoxylin–eosin staining of lung‐metastatic nodules in model intracardiac‐injected with shN and vector cells. Scale bars, 200 μm. (I) Overview of cisplatin‐ or doxorubicin HCl‐treated tumours in xenograft mice model subcutaneously implanted with shN and vector cells. NS indicates normal saline, as control. (J) Growth curves of cisplatin‐ or doxorubicin HCl‐treated tumour volumes formed by shN and vector cells. Data were presented as the mean ± SEM. (K) Haematoxylin–eosin and IHC staining of drug‐treated tumours in xenograft mice model subcutaneously implanted with shN and vector cells. Scale bars, 200 μm.

A xenograft mouse model was established to elucidate the role of NSUN2 in ATC progression in vivo (Figures 4A and B). Tumours harvested from shN cells‐injected mice showed significantly slower growth and reduced sizes and weights than those injected with vector ATC cells (Figures 4B and C and S3A and B). IHC staining indicated reduced Ki67 staining in tumours from shN‐1 and shN‐2 groups, confirming that NSUN2 inhibition reduced ATC proliferation in vivo (Figures 4D and E and S3C). The lung metastasis mice model, intracardiac‐injected with NSUN2 knockdown and control KHM‐5 M showed that shN ATC cells developed fewer metastatic nodules (Figure 4G). Haematoxylin–eosin (H&E) staining revealed that NSUN2 knockdown alleviated alveolar tissue destruction by ATC cells (Figures 4H and S3E). Under treatment with doxorubicin HCl or cisplatin, tumour volumes and weights were significantly lower in the shN group than those in the control group (Figures 4J and S3F and G). Furthermore, NSUN2 knockdown notably improved the drug efficacy (Figure 4I). After drug administration, the shN group's H&E staining showed more necrotic foci (Figure 4K, left panel). Induction of p53 was detectable in all samples (Figure 4K, right panel). It was, however, noteworthy that after the drug treatment, the P53 staining in the shN group was lightened. This phenomenon may be associated with the apoptosis‐induced decrease of cells. Therefore, NSUN2 knockdown reduced the proliferation and metastasis, whereas, with treatment with both drugs, the in vivo survival of ATC cells was further reduced.

### 2.5 NSUN2 regulates tRNA m^5^C modification and facilitates global translation in ATC

**FIGURE 5:**
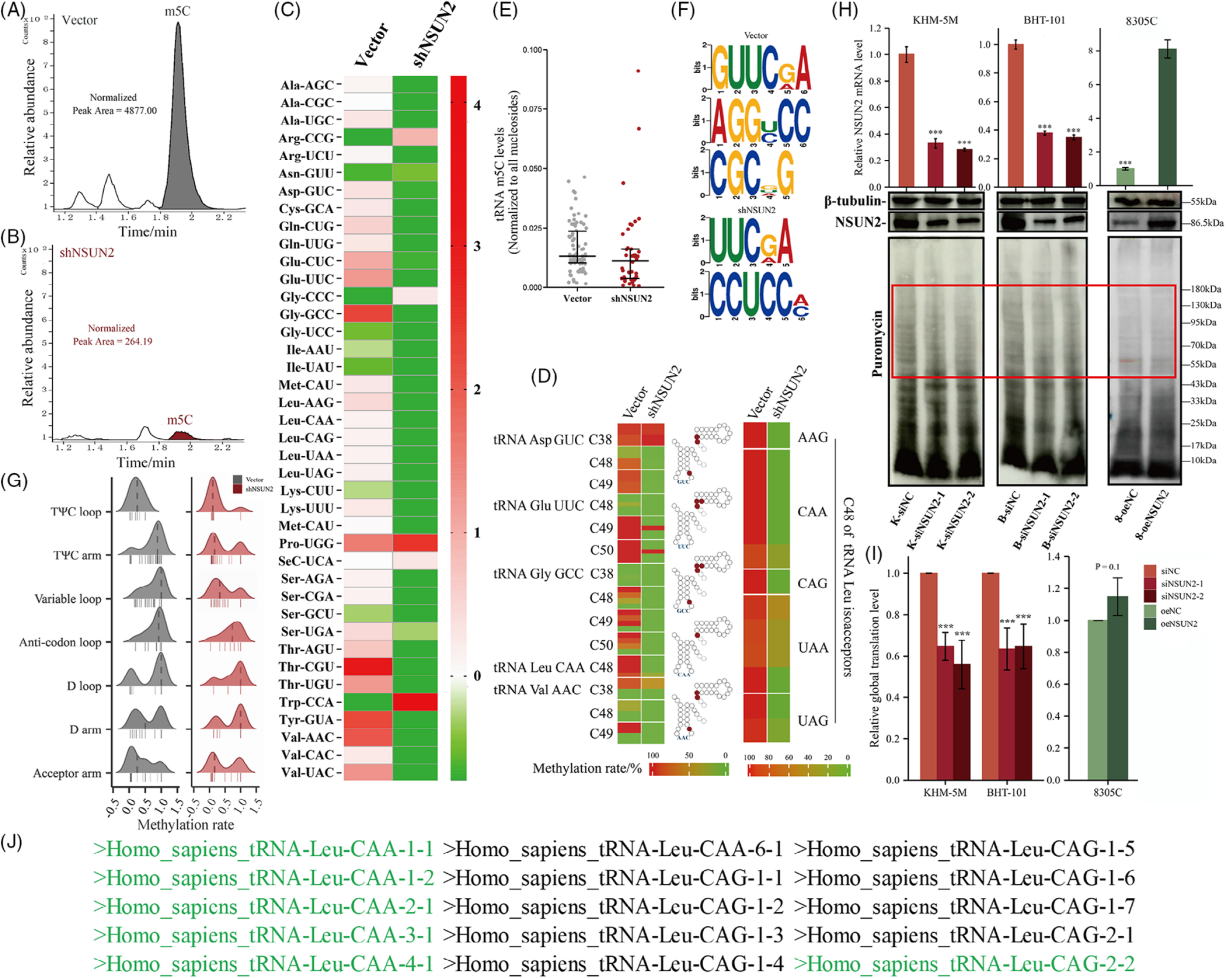
The deletion of cytoplasmic tRNA methylation induced by NSUN2 knockdown showed a species bias and secondary structure bias, and led to a decrease in the overall protein translation level. (A and B) LC–MS analysis of total 5‐methylcytidine (m^5^C) levels in purified tRNA from vector (A) and shN (B) cells. (C) Heatmap of m^5^C‐modified cytoplasmic tRNAs in the NSUN2 knockdown and control cells. Each cell shows the summarised m^5^C level of a representative tRNA isodecoder. The colour represents the relative intensity of methylation changes. (D) Heatmap illustrating the methylation levels of different cytosines on candidate tRNAs, as determined by bisulphite sequencing of tRNA preparations from the NSUN2 knockdown and control cells. A gradient from green to red indicates the methylation rate from 0 to 100%. (E) Quantification of m^5^C level on m^5^C‐modified tRNAs. (F) Motif sequence at m^5^C sites, *p* value < .001, *E* value < 0.01. (G) Ridge plot showing the change of m^5^C methylation rate density on tRNA secondary structure between vector and shN cells. Each short vertical line represents methylation rate of a specific m^5^C site. (H) The effects of NSUN2 knockdown and expression on protein synthesis in KHM‐5 M, BHT‐101 and 8305C as analysed by puromycin intake assay. (I) Statistical analysis on the basis of puromycin incorporation to monitor protein synthesis. Data are shown as mean ± SD of *N* = 3 biological replicates. (J) Human genome mature tRNA Leu‐CAA and CAG, the green part indicates that there are detectable m^5^C modification sites on the tRNA sequence.

Because NSUN2 has been shown to methylate tRNAs,^16^, ^29^, ^30^, ^31^, ^32^ LC–MS was used to analyse total 5‐methylcytidine levels in purified tRNA preparations from vector and shN KHM‐5 M cells. The result demonstrated global loss (fold change = 18.41) of tRNA cytosine‐C5 methylation in shN cells (Figures 5A and B). For a comprehensive understanding, ATC's tRNA m5C methylation pattern was assessed using RNA bisulphite sequencing.^33^ The residual 5‐methylcytidines occurred in different tRNA iso‐acceptors and iso‐decoders (Figure S4A). During NSUN2 knockdown, most tRNAs experienced a substantial reduction in m^5^C levels (Figures 5C and E and S4A and B). Several sites, especially C48 and C49, in the known NSUN2 substrates^9^, ^16^ including tRNA‐Leu‐CAA, ‐Glu‐UUC, ‐Gly‐GCC and ‐Val‐AAC completely lost their m^5^C modifications (Figure 5D). They are consistent with Trm4‐dependent tRNA methylation patterns described in yeast.^34^ Among these, five of six leucine tRNA Leu CAA iso‐acceptors were affected (Figures 5D and J). Therefore, motif analysis was performed to elucidate enriched m^5^C sites in tRNA sequences (Figure 5F). Furthermore, tRNA m^5^C patterns mediated by NSUN2 on secondary tRNA structure were characterised. The shifts in methylation levels are most likely to appear in the variable loop and TΨC arm (Figure 5G), indicating that they are sensitive to NSUN2 knockdown.

Considering the primary function of tRNAs, it was we hypothesised that hypomethylated tRNAs may slow down the global translation. This hypothesis was tested through puromycin intake assays,^35^ which elucidated global protein synthesis in NSUN2 knockdown or overexpressed cells. NSUN2 knockdown resulted in decreased global protein synthesis in KHM‐5 M and BHT‐101 cell lines, which could be slightly rescued by overexpressing, as evidenced in NSUN2 overexpressed 8305C (*p* = .1) (Figures 5H and I and S5). These results further supported the role of tRNA m^5^C in promoting protein synthesis, implying that NSUN2 might regulate translation through tRNA epigenetic modification.

### 2.6 NSUN2 regulates the translation of pro‐oncogenic mRNAs that contribute to ATC progression

**FIGURE 6:**
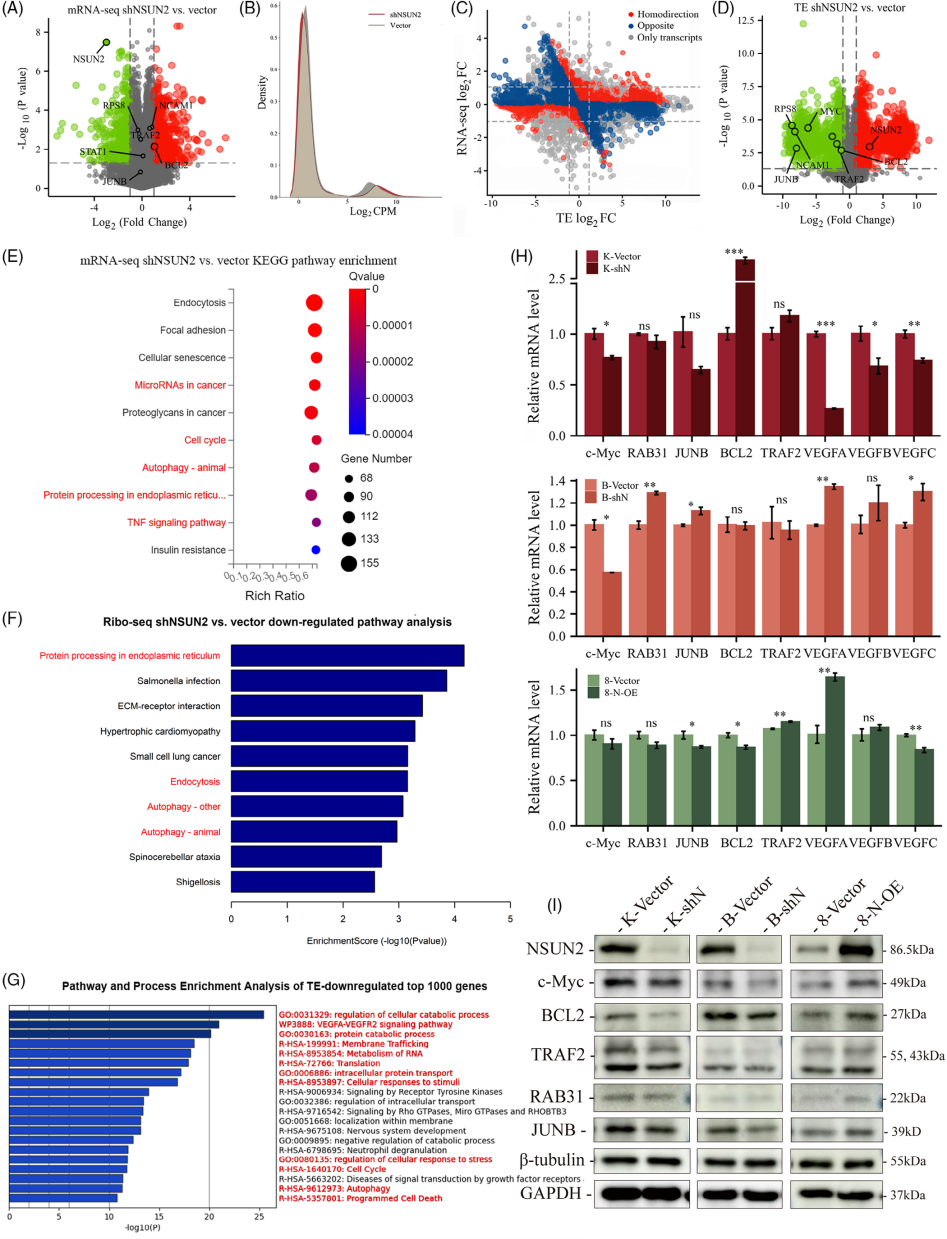
The transcriptomic and translational changes caused by NSUN2 deletion are mainly concentrated in the tumourigenesis and development‐related pathways, and NSUN2 knockdown mechanically leads to codon‐dependent translation attenuation. (A) Volcano plots of mRNA‐seq for the differences in vector and shN groups determined by *t*‐test. The *y* axis indicates the *p* values. The *x* axis indicates fold change (FC). The significantly down‐regulated (log_2_FC ≤−1, *p* < .05, green) or up‐regulated (log_2_FC ≥1, *p* < .05, red) genes were shown. Vertical dashed lines indicate cut‐off of log_2_FC (1 or −1); horizontal dashed lines indicate cut‐off of *p* value (.05). (B) Density plot showing translation changes upon NSUN2 knockdown by Ribo‐seq. (C) Scatterplot of the FCs of TE and mRNA abundance in NSUN2‐depleted KHM‐5 M (log_2_FC ≥ 1 and ≤−1; *p* < .05). Homo‐direction and opposite are used to describe the relative trend of mRNA level and translation level. (D) Volcano plots of TE differences in vector and shN groups. The *y* axis indicates the *p* values. The *x* axis indicates fold change (FC). The significantly TE‐down‐regulated (log_2_FC ≤−1, *p* < .05) or up‐regulated (log_2_FC ≥1, *p* < .05) genes were shown. Vertical dashed lines indicate cut‐off of log_2_FC (1 or −1); horizontal dashed lines indicate cut‐off of *p* value (.05). Representative genes were marked. (E) KEGG enrichment analysis for mRNA‐seq between shN and vector. (F) Pathway analysis for TE‐down‐regulated genes. (G) Pathway and process enrichment analysis of top 1000 TE‐down‐regulated genes. (H) qRT‐PCR analysis of representative TE‐down‐regulated genes. (I) Immunoblot detection of representative TE‐down‐regulated genes.

**FIGURE 7:**
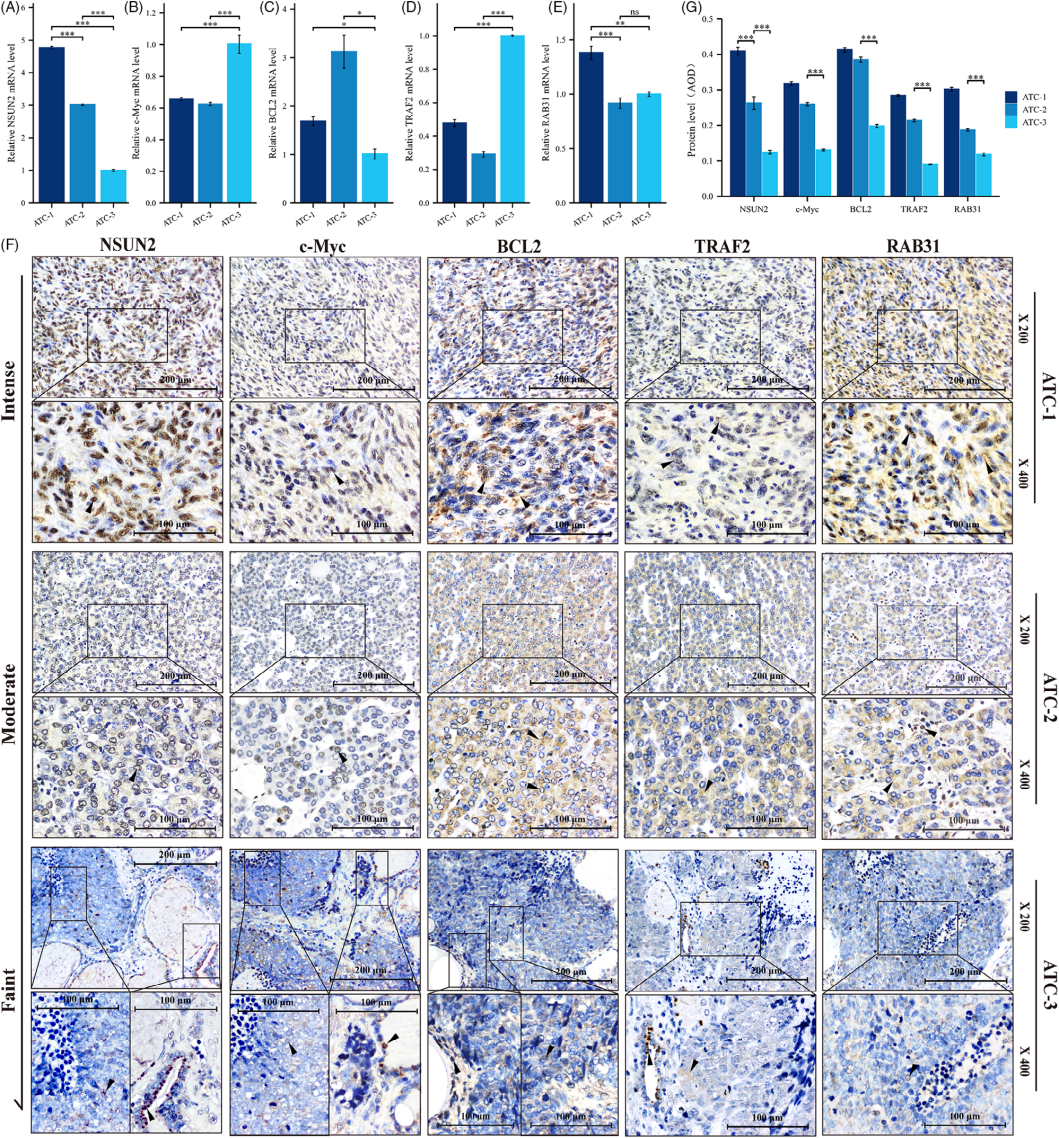
The correlation between NSUN2 and the expression of representative genes was also verified in ATC tissues. (A–E) qRT‐PCR analysis showed that there was no obvious correlation between c‐Myc (B), TRAF2 (C), BCL2 (D), RAB31 (E) and NSUN2 (A) mRNA levels in ATC tissues. (F) The ATC tissues were arranged according to the expression level of NSUN2 protein, and those with low expression of NSUN2 tended to have lower protein levels of c‐Myc, TRAF2 and RAB31. (G) Quantification of IHC staining in ATC tissue by average optical density (AOD), ANOVA.

Cancers demonstrate aberrant translational reprogramming.^36^ Compared with DTC, ATC showed up‐regulation of certain pathways.^37^ Therefore, to investigate what specific mRNA subgroups were regulated by NSUN2, vector and shN cells were subjected to mRNA and ribosome profiling. mRNA‐seq showed that differentially expressed transcripts between vector and shN cells were enriched in pathways related to protein processing in the endoplasmic reticulum, microRNA in cancer, cell cycle and TNF signalling pathway (Figures 6A and E and S4D). The ribosome‐seq indicated that NSUN2 knockdown significantly altered gene translation levels in ATC cells (Figure 6B). The decreased‐translated genes were associated with pathways involved in protein processing in the endoplasmic reticulum, autophagy and endocytosis (Figures 6F and S4E). Translation efficiency (TE) is the ratio of normalised translation to transcription levels. It was revealed that the TE of the mRNA subset changed after NSUN2 knockdown (Figure 6C), yielding a set of 3837 genes with decreased TE (Figure 6D). The top 1000 genes with decreased TE enriched in several cancer‐related pathways, including metabolism of RNA, translation, regulation of cellular response to stress and VEGFA–VEGFR signalling pathway (Figure 6G), suggesting the role of NSUN2 in a crucial pro‐cancer program. Different candidate genes related to ATC progression were then selected to verify the NSUN2's role in promoting cancer‐related translation. A significant decrease in proto‐oncogenic genes, including JUNB, TRAF2 and RAB31, was observed in translation (Figures 6D and I), while their mRNA levels were out of sync with, or even reversed from changes in protein levels, before and after NSUN2 knockdown (Figures 6A and H). These genes were further validated in NSUN2‐overexpression 8305C cells (Figure 6I), which indicated that NSUN2 promotes translation. Intriguingly, NSUN2 inhibition decreased the protein level of BCL2, despite a doubling of its mRNA level in KHM‐5 M cells (Figure 6H). Furthermore, NSUN2 knockdown reduced mRNA and TE levels of c‐Myc (Figures 6A and D). However, the reduction in mRNA level cannot explain the dramatic decline in TE of c‐Myc [Figures 6A and D; shN vs. vector log_2_(Fold change): mRNA −0.095, *p* = .01, TE −6.23, *p* < .01)]. In the ATC tissue samples, compared with those with low NSUN2 expression, higher NSUN2 expression could maintain higher protein expression, such as c‐Myc, TRAF2 and RAB31, despite lower mRNA levels (Figure 7). These data showed that oncogenic NSUN2 regulates the translation of a subset of mRNAs that are critical for ATC anti‐apoptosis and development.

### 2.7 Some TE‐down‐regulated tumour‐related genes showed codon usage bias

**FIGURE 8:**
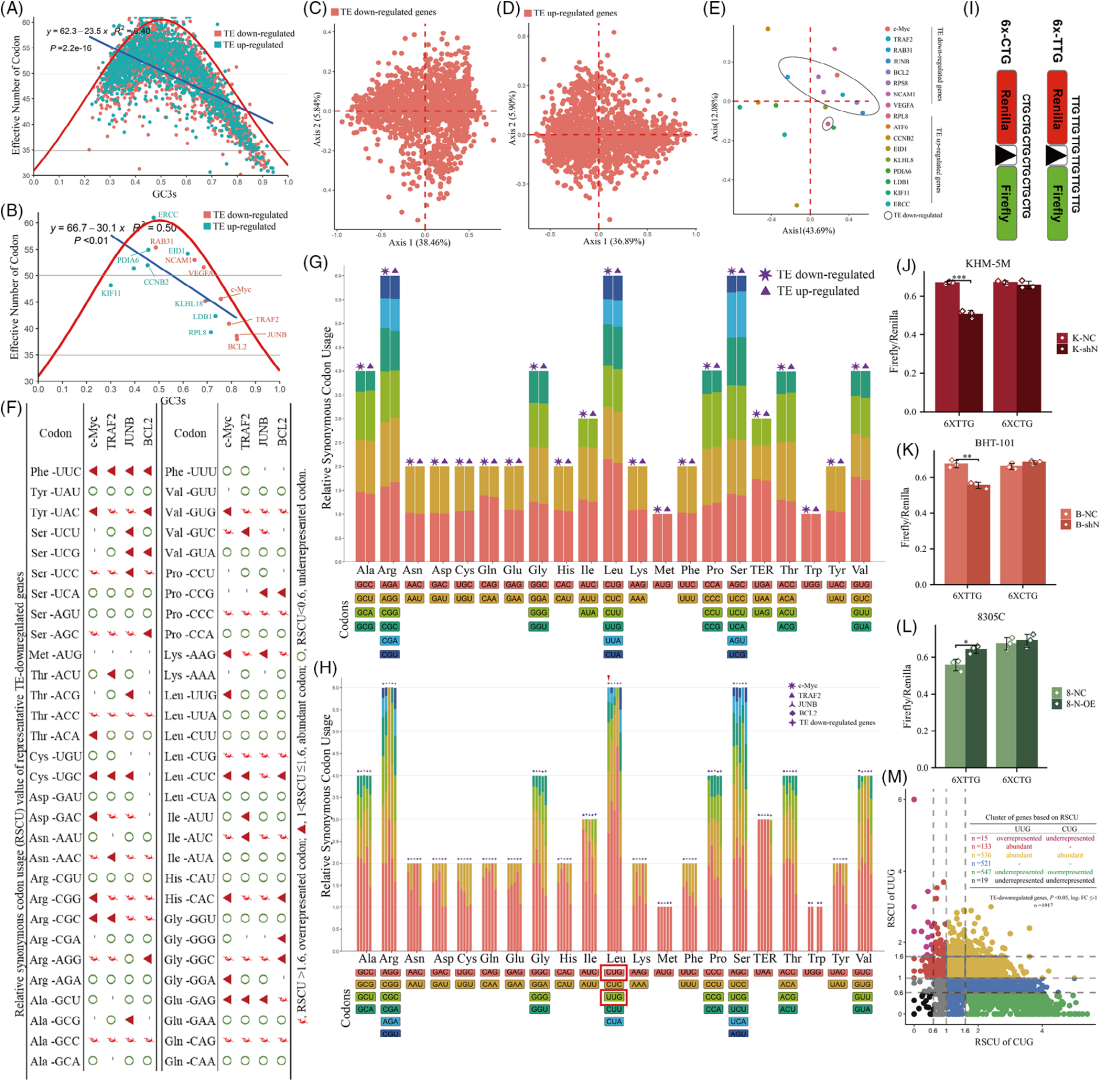
Some TE‐down‐regulated genes showed codon usage bias. (A and B) ENC‐plot analysis of significantly TE‐changed (|log_2_FC| ≥1, *p* < .05) genes (A) and representative genes (B). The red curve was the expected ENC‐values versus GC_3s_. ENC, effective number of codons. GC_3s_, GC content in the third digit of the synonymous codon. (C–E) Correspondence analysis of TE‐down‐regulated (C), TE‐up‐regulated (D) and representative (E) genes. (A plot with Axis1 against Axis 2 was plotted based on RSCU values of these genes.) (F) The table lists the RSCU values for each codon of four representative TE‐down‐regulated genes. ¢ (RSCU > 1.6), overrepresented codon; ▲ (1 < RSCU ≤ 1.6), abundant codon; ○ (RSCU < .6), underrepresented codon. (G) Relative synonymous codon usage (RSCU) values of top 1000 TE‐down‐regulated and TE‐up‐regulated genes. (H) Comparison of RSCU values of TE‐down‐regulated genes as a whole and four representative genes alone. (I) Scheme illustrating the dual luciferase reporter system for assessing the TE of TTG‐rich protein in vector and shN ATC cells. (J–L) 6X‐TTG and 6X‐CTG reporter activity was quantified in vector and (J and K) shN ATC cells or (L) N‐OE cells. Data represent mean ± deviation for three biological replicates. (M) According to the RSCU values of UUG and CUG, the TE‐down‐regulated genes were divided into different clusters. The colour of dots is consistent with the font colour in the table. RSCU > 1.6, overrepresented; RSCU < .6, underrepresented; 1 < RSCU ≤ 1.6, abundant.

First, the CDS sequences codon usage characteristics of genes with significant TE changes were explored. The effective number of codons (ENC) values in TE‐down‐regulated and ‐up‐regulated genes were clustered around 45−60, with most sequences located below the standard curve, supporting that codon usage bias was mainly affected by natural selection during evolution (Figure 8A). However, a few representative genes indicated ENC values clustered around 35−47.5 (Figure 8B), indicating acceptable codon bias in these sequences. Furthermore, in the correspondence analysis (COA) based on 59 synonymous codons’ relative synonymous codon usage (RSCU) values, the first axis represents the highly correlated main contributor to the codon bias pattern. In TE‐down‐regulated genes, the first two main axes accounted for 38.46 and 5.84%, respectively (Figure 8C). In TE‐up‐regulated genes, the first two main axes can explain 36.89 and 5.90% of the COA result (Figure 8D), respectively. There was a different data accumulation between TE‐down‐regulated and ‐up‐regulated genes (Figures 8C and D), indicating inconsistent formation of codon bias in these two subgroup sequences. Several representative genes selected in this study retain this characteristic (Figure 8E). RSCU values of all sequences were counted and shown in a bar chart to further reveal the codon usage pattern. There was no obvious difference in the RSCU values of TE‐up‐regulated or down‐regulated genes through concatenate analysis (Figure 8G). However, some codons in genes, including c‐Myc, TRAF2, JUNB and BCL2, had significantly higher than average RSCU values (Figure 8H). The result showed that the abundant codons in these 4 genes were GCC, CGC, CGG, AAC, ACC, AGC, UCC, UAC, UUC, GUG, GUC, CCC, AAG, CUG, CUC, AUC, CAC, GGC, GAG and CAG (Figure 8F). As Figure 5D showed, tRNA Gly‐GCC and tRNA Leu‐CAG which was complementary to GGC and CUG, have detectable m^5^C sites at the variable loop. Additionally, codon UUG was abundant in c‐Myc (Figures 8F and H).

As indicated before, the reduction in mRNA level cannot explain the dramatic decline in TE of c‐Myc (Figures 6A and D). It was revealed that upon NSUN2 knockdown, the single‐site methylation at the variable loop of tRNA Leu‐CAA was almost completely deleted, while the same‐position methylation of tRNA‐Leu‐CAG was not significantly affected (Figures 5D and J). Moreover, the abundant codon CUG and UUG (complementary to CUG and UUG, respectively) in c‐Myc were also assessed. It was hypothesised that loss of tRNA m^5^C modification would reduce the TE of proteins rich in relevant codons. To test TE changes in shN cells caused by m^5^C depletion in tRNA Leu‐CAA, a dual‐luciferase reporter construct was designed (Figure 8I), where the linker region between these the two coding sequences was either six CTG (as control) or six TTG codons in a row (6X‐TTG and 6X‐CTG, respectively). These two Leu‐containing tRNA‐complementary codons coded sequences could minimise the error caused by the cell's utilisation of different amino acids and reduce the interference caused by the wobble‐site modification crosstalk.^38^ The NSUN2 inhibition significantly decreased 6X‐TTG reporter expression compared with vector cells (Figures 8J and K), whereas its overexpression increased TE of 6X‐TTG (Figure 8L). Such effects were not observed for the 6X‐CTG reporter (Figures 8J–L). Coupled with the evidence for decreased m^5^C modification at the variable loop of tRNA (Figure 5D), the data indicated that NSUN2‐catalysed m^5^C incorporation in tRNA Leu‐CAA enhanced the translation of UUG‐rich mRNA. Retrospectively, differences in m^5^C methylation between tRNA Leu‐CAA and Leu‐CAG could explain 35.7% [(133 + 15 + 536)/1917] of TE‐down‐regulation due to UUG codon bias (Figure 8M).

### 2.8 Knockdown of NSUN2 reversed the detrimental c‐Myc to NSUN2 cycle

**FIGURE 9:**
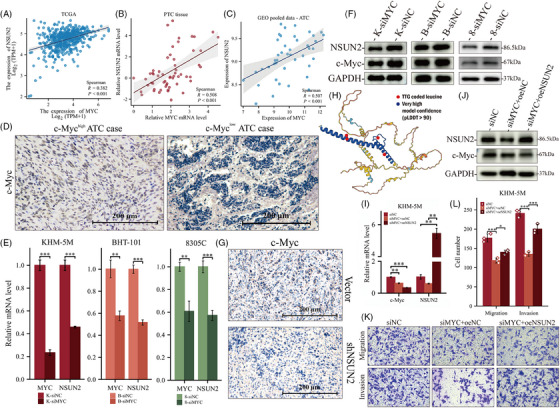
NSUN2 levels are regulated by c‐Myc, and the malignancy caused by c‐Myc can be partly performed by NSUN2. (A–C) Association between c‐Myc and NSUN2 in thyroid cancer. (A) PTC RNA‐seq data obtained from TCGA database. (B) PTC patients’ tissue from Xiangya Hospital. (C) ATC RNA‐seq pooled data from GSE33630, GES65144 and GSE76039. (D) IHC staining of c‐Myc in ATC tissue derived from Figure 1D. Scale bars, 200 μm. (E and F) qRT‐PCR (E) and WB (F) validated NSUN2 was down‐regulated upon c‐Myc knockdown in all ATC cell lines used in this article. (G) IHC staining of c‐Myc in tumours samples in xenograft mice model from Figure 4. Scale bars, 200 μm. (H) Three‐dimensional structure from AlphaFold (predicted) for c‐Myc protein. The blue part indicates very high model confidence (pLDDT > 90). The red dots indicate leucine encoded by TTG in c‐Myc protein. (I and J) Validation of c‐Myc knockdown and NSUN2 overexpression for rescue assay. (K and L) Transwell assay (K) and statistical analysis (L) showing that overexpression of NSUN2 partly rescued the decreased migration and invasion upon c‐Myc knockdown.

c‐Myc is an upstream transcription factor of NSUN2^19^ (Figure S4J). To verify the effect of c‐Myc on NSUN2 in ATC (Figure 9D), the correlation between NSUN2 and c‐Myc expression levels was examined. Data from TCGA and quantitative reverse‐transcription polymerase chain reaction (qRT‐PCR) analysis of PTC patients showed a positive correlation between NSUN2 and c‐Myc mRNA levels (Figures 9A and B). Furthermore, c‐Myc knockdown significantly reduced NSUN2 mRNA and protein levels compared with control cells (Figures 9E, F and G). The rescue assay revealed that NSUN2 overexpression (Figures 9I and J) rescued the weakened invasion and migration abilities of KHM‐5 M cells caused by c‐Myc knockdown (Figures 9K and L). Thus, regardless of the pathways between them, c‐Myc in ATC is the major upstream regulator of NSUN2. It has been indicated that c‐Myc has a leucine zipper dimerisation domain, which is required for efficient DNA binding,^39^, ^40^, ^41^, ^42^ and in this domain, most leucine residues are encoded by TTG on the c‐Myc gene (Figure 9H).The leucine residues in aligned coiled‐coil structures of c‐Myc are essential for its function.^43^, ^44^ Nakajima and colleagues^45^ revealed that a single amino acid substitution in this domain inactivates the transforming ability of the N‐myc gene product. Furthermore, mutations in the leucine‐zipper structure also cause functional inactivation in c‐Myc.^46^ Consistent with the high‐frequency codon reporter assay data (Figure 8F), the c‐Myc function requires the correct translation of TTG‐coded leucine in its zinc finger structure, suggesting that NSUN2 knockdown caused an early decrease in NSUN2‐mediated m^5^C in tRNA Leu‐CAA, which would lead to impaired translation of TTG‐rich leucine‐zipper in the upstream transcription factor c‐Myc.

### 2.9 m^5^C modification mediated by NSUN2 functions in the stability of tRNA

**FIGURE 10:**
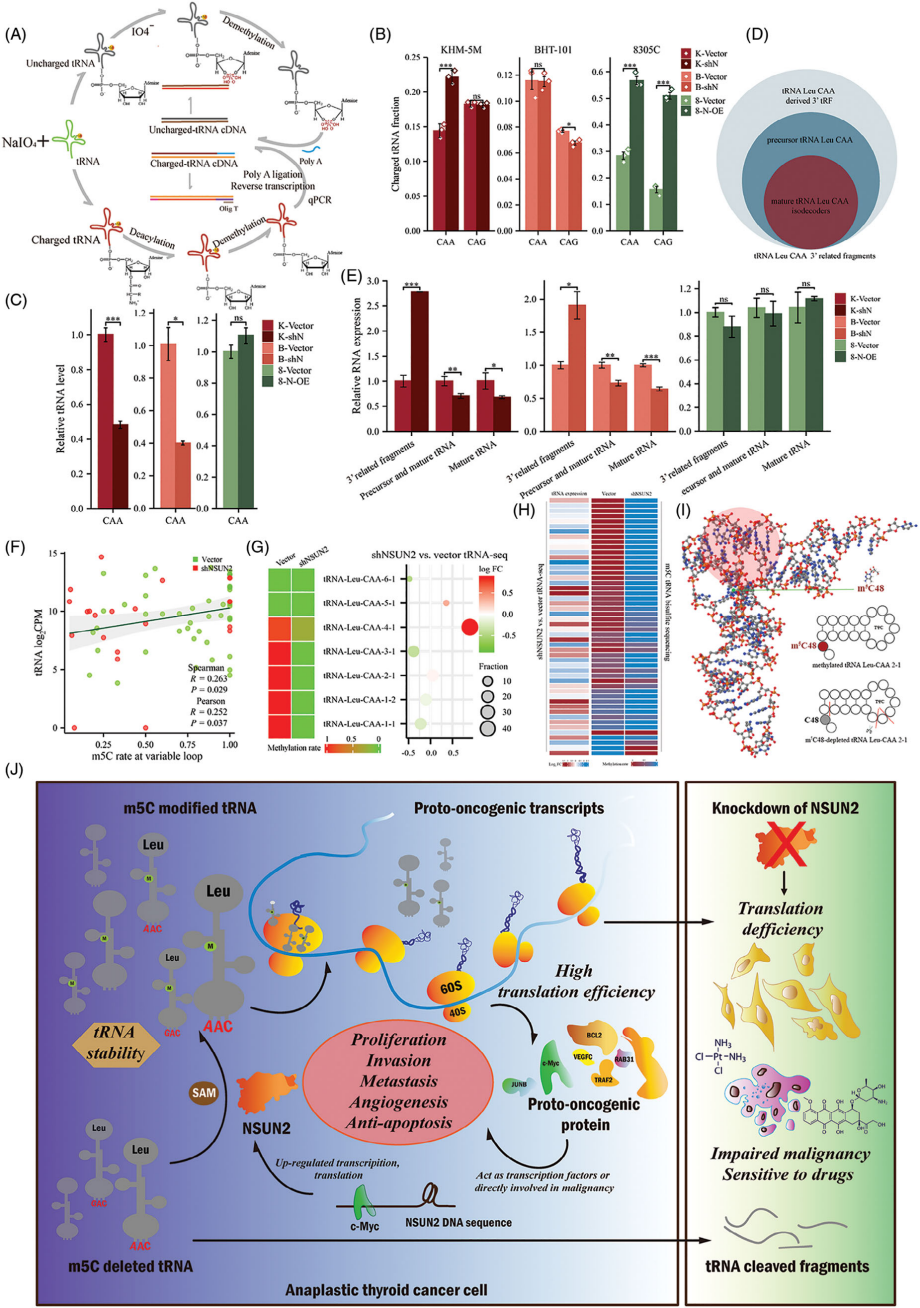
Depletion of NSUN2‐mediated m5C caused a decrease in tRNA and an increase in cleaved fragments. (A) A method for tRNA charging assay. (B) tRNA charging level of indicated isodecoder family was measured. Primers specific to Leu‐CAA and Leu‐CAG were used. (C) qRT‐PCR analysis of tRNA Leu‐CAA. (D) Relationship of tRNA related RNA fragments. (E) qRT‐PCR analysis of tRNA Leu related RNA fragments. (F) Correlation scatter plot showing that tRNA expression is positively associated with m^5^C modification at variable loop. (G) Comparison between tRNA Leu‐CAA isodecoders expression and m^5^C modification. The bubble size represents the proportion of tRNA Leu‐CAA isodecoders in vector cell. (H) Comparison between tRNA expression and m^5^C modification at variable loop. Each row represents the same tRNA isodecoder. (I) The 3D structure diagram shows the position of C48 (green dot) and TΨC loop/arm (red shaded part) in tRNA Leu‐CAA‐2‐1. The blue, red, white orange and grey sphere represents the nitrogen atom (*N*), oxygen atom (*O*), hydrogen atom (*H*), phosphorus atom (*P*) and the carbon atom (*C*). In the secondary structure of tRNA Leu‐CAA, short red line indicates the possible cleavage sites of tRNA after methylation deletion. (J) Working model for NSUN2 mediated tRNA m^5^C modification in regulation of ATC tumourigenesis. NSUN2 sustains tRNA stability by catalysing m^5^C modification and enhances translation in cancer.

The above data raised a question: how does aberrant m^5^C modification affect the availability of tRNA? This was addressed by first assessing the charging status of tRNA in vector and shN samples. The sodium periodate oxidation method followed by ligating at the 3′ end poly‐A tail of tRNA was performed to quantify charged‐tRNA (Figure 10A). After periodate treatment, ─OH and subsequently the ribose ring on the 3′ end of uncharged tRNA were destroyed, while the 3′ end of charged tRNA was protected by amino acid attached to it and remained functional. Although the aminoacylation level cannot fully explain the decreased global translation, surprisingly, increased charged tRNA was observed in NSUN2‐overexpressed cells (Figure 10B). It has been reported that m^5^C protected tRNA from angiogenin‐mediated cleavage induced by arsenite, thermal stimulation or ultraviolet irradiation.^47^, ^48^ Furthermore, full sequences of mature tRNA Leu‐CAA and related cleaved fragments (Figures 10D and S4I) were compared, and several primers were designed to quantify tRNA products in cells (Figures 4F–I and Table S2). The lack of NSUN2 decreased tRNA Leu‐CAA reserves and increased at least three sets of 3′‐tRF fragments (Figures 10C and E and S4I). However, NSUN2 overexpression prevented the cleavage of tRNA in 8305C cells (Figure 10E). Next, the cleavage sites of these possible fragments on tRNA Leu‐CAA were assessed (Figure 10I), which revealed that these sites were close to C48 in the three‐dimensional structure of tRNA Leu‐CAA 2‐1 (Figure 10I), suggesting that the absence of m^5^C may lead to increased accessibility of lyase to tRNA sites,^49^ similar to the mechanism by which angiogenin cuts the anticodon loop.^47^, ^48^ Moreover, tRNA sequencing further confirmed the correlation between m^5^C modification and tRNA expression level. After NSUN2 knockdown, the expression of tRNA Leu‐CAA 4‐1 with a small amount of methylation remained increased, while other m^5^C‐deleted tRNA Leu‐CAA decreased (Figure 10G). Additionally, the tRNA expression and m^5^C level were positively correlated (Figures 10F and H). Altogether, these data supported the link between tRNA m^5^C modification and tRNA stability.

## 3 DISCUSSION

Other than their involvement in genetic decoding, tRNAs have recently been indicated to be crucial for cell homeostasis.^24^, ^50^, ^51^, ^52^, ^53^ With >90 annotated modified nucleoside structures in humans,^54^ most RNA modifications have been identified in tRNAs. This binary event expanded the composition of tRNA molecules from four standard nucleosides to over 160 differently modified nucleosides. Certain modifications affect the folding or the stability of tRNA tertiary structure,^55^, ^56^ the efficiency and accuracy of translation,^57^ and the tRNA recognition specificity by other interacting proteins including elongation factors, aminoacyl‐tRNA synthetase and RNases^58^, ^59^, ^60^; It has been indicated that aberrant tRNA modifications are significantly associated with cancer.^61^, ^62^ This research identified NSUN2, a tRNA cytosine C‐(5)‐methyltransferase,^32^, ^63^, ^64^ as a tumourigenic factor in ATC. NSUN2 down‐regulation suppressed tumour formation, proliferation, migration, invasion and lung metastasis in ATC. Moreover, NSUN2 was found to confer resistance to chemotherapy drugs in ATC, suggesting that NSUN2 regulates a tumour progression network. Furthermore, the identification of NSUN2's function in translation regulation highlights the diverse mechanisms of tRNA m^5^C modification in tumourigenesis.

When compared with DTC, ATC has intricated heterogeneity, including genomic and epigenetic abnormalities.^65^ Classical molecular pathology of ATC includes the mutation of BRAF^V600E^, Ras, ALK and increase in the gene amplification or copy number of PI3KCA, EGFR, VEGFR1/2, Kit, Met, Ras, BRAF and so on.^37^ The activation of these multiple oncogenes or related pathways including c‐Myc, PI3K and AKT drives expression of ribosomal RNA, ribosomal protein genes and other specific oncogenes.^6^, ^66^ Here, NSUN2 suppression reduced global translation and protein expression of candidate proto‐oncogenic genes, including c‐Myc, RAB31, JUNB, TRAF2 and BCL2. The down‐regulation of these anti‐apoptotic proteins might sensitise the ATC cells to genotoxic anti‐tumour drugs. Surprisingly, since NSUN2 maintains mRNA and translation levels of its transcription factor c‐Myc, its knockdown inhibits this detrimental circle. c‐Myc's interaction with multiple ATC‐related pathways has made it increasingly central to cancer research (Figure S4J). It has been reported that EIF1AX and RAS mutations synergistically promote thyroid tumourigenesis via ATF4 and c‐Myc, which were associated with an increased abundance of glutamine and leucine transporters.^67^ TERT is the direct target gene of c‐Myc and regulates the stability of c‐Myc in cancer.^68^ The co‐signalling pathway of KRAS and thyroid hormone receptor β mutants promotes the development of ATC through c‐Myc up‐regulation.^69^ The c‐Myc targeting drug, JQ1, has exhibited prominent effects against ATC in vitro.^70^, ^71^ This research provides a novel target for inhibiting ATC initiation and progression.

This study revealed a significant reduction in m^5^C modification at position C48 of tRNA Leu‐CAA upon NSUN2 inhibition, consistent with the literature demonstrating that tRNA Leu‐CAA was a specific substrate for NSUN2.^16^ Interestingly, some tRNAs indicated increased m^5^C modification levels after NSUN2 knockdown (Figures 5C and S4A), and this could be explained by the complementary roles of DNMT2 and NSUN2 in tRNA modification,^9^ where the absence of NSUN2 causes a compensatory increase in DNMT2‐mediated tRNA m^5^C modification for maximum maintenance of homeostasis. Furthermore, NSUN2 reduction also affected the expression of tRNA Leu‐CAA, validating that unmethylated tRNAs, at least at the variable loop, are less stable and more prone to tRNA fragmentation.^24^ tRNA modification causes domain rearrangement while providing tRNA rigidity and elasticity to maintain optimal translation activities.^55^ When m^5^C is deficient, tRNA more readily interacts with its cleavage enzymes,^49^ or impaired translation may induce a stress response at the expense of tRNA cleavage.^72^, ^73^ As an essential branched‐chain amino acid, Leucine is a signalling molecule regulating metabolism, immunity and protein synthesis via a special signalling network, especially PI3K/AKT/mTOR signal pathway.^74^, ^75^, ^76^ Currently, the molecular basis of leucine involvement in protein synthesis mainly includes leucine‐rich repeat sequence motif and leucine zipper structure. The leucine‐rich proteins, including small leucine‐rich proteoglycans, LRRC15 and basic leucine zipper transcription factor, NRF2, have emerged as novel therapeutic targets against cancer.^77^, ^78^, ^79^ This study proposes that differences in the TE of tRNA Leu‐CAG and Leu‐CAA may cause codon‐biased translation in ATC cells. That is, for maintaining malignant translation reprogramming, tumour cells require more stable tRNA Leu‐CAA than Leu‐CAG. This result is consistent with the role of tRNA Leu‐CAG in the translation of tumour suppressors.^80^

Intriguingly, more 3′tRFs derived from m^5^C‐deleted tRNA Leu‐CAA were also observed; this discovery introduced a significant complexity to tRNA fragmentation events. Previous studies have linked tRF to stress‐induced translation inhibition.^81^, ^82^ Meanwhile, tRF affects TE by regulating ribosomal protein translation.^83^, ^84^ Furthermore, numerous studies have indicated anti‐ or proto‐tumour properties of tRF.^85^, ^86^, ^87^, ^88^, ^89^, ^90^ Further research is warranted to examine the regulatory effects of these 3′tRFs, particularly during stress. The changes in oncogenic translation and tRNA aminoacylation levels after m^5^C deletion observed in this investigation did not consistently align across cell lines. This discrepancy warrants further investigation because of the potential variations in cell lines used, including factors such as sex.^91^, ^92^

In addition to the proteins identified as downstream of NSUN2‐dependent proteomic regulation in ATC, a group of non‐coding RNAs, including but not limited to tRNA Leu‐CAA and its derived fragments, were identified, which may govern additional crosstalk and phenotypes. Future studies are needed to explore unanticipated roles for NSUN2‐dependent tRNA modification regulation in homeostatic and metabolic regulation. More broadly, this study motivates further research on tRNA methylation as a regulator of protein synthesis and tumourigenesis.

## 4 CONCLUSIONS

In conclusion, this study identified that cytosine‐C5 methyltransferase NSUN2, which catalyses most of tRNA, is vital for sustaining the high tRNA stability demands in translation reprogramming in ATC. Furthermore, high NSUN2 expression in ATC cells enhances the translation of key transcription factors and anti‐apoptosis related genes to promote tumour progression and resistance. Suggesting that it can serve as a target for codon‐dependent pro‐cancer translational programs. This research indicated that m^5^C methyltransferase modulates tRNA stability, expression of its downstream oncogenes and downstream codon‐dependent oncogenic translation network that enhances ATC initiation and growth. Furthermore, this study provides new opportunities for targeting the translation reprogramming, malignant transformation and drug resistance in cancer cells.

## 5 METHODS AND MATERIALS

### 5.1 Patient samples

All the ATC and PTC samples were obtained from Xiangya Hospital Central South University and approved by Xiangya Hospital's Protection of Human Subjects Committee (No. 202004192) with informed consent from patients. Some of the specimens were gifts from colleagues in thyroid surgery department. Paraffine ATC samples from patients were used under institutional review board‐approved protocols.

### 5.2 Bioinformatics analysis

Bioinformatics analysis and visualisation were performed by R (version 4.3.1). Use the sva package's ComBat function to remove batch differences. Use the limma package [3.52.2] to analyse the difference between two groups. GSE76039, GSE65144, GSE29265 and GSE33630 data were obtained from GEO database through GEOquery [2.64.2] package, then normalised again through the limma package's normalizeBetweenArrays function, and then the limma package is used for difference analysis between two groups.

### 5.3 Cell culture

Nthy‐ori‐3‐1, BHT‐101 (from female), ^91^ 8305C ^93^ and KHM‐5 M (from male)^92^ cell lines purchased from American Type Culture Collection were authenticated by STR profiling. All cells were cultured in RPMI‐1640 (Gibco) supplemented with 10% foetal bovine serum (FBS; Gibco), 1% streptomycin and penicillin (Gibco). Cells were kept in a properly humid atmosphere with 5% CO_2_ at 37°C in an incubator (Thermo Scientific).

### 5.4 siRNA knockdown and lentiviral transduction

Small interfering RNAs (siRNAs) targeting NSUN2 and c‐MYC (Table S2) were directly synthesised by ZORIN Biotechnology Co. siRNA transfection was conducted as previously reported.^94^ Lentivirus vectors of NSUN2 knockdown and overexpression were purchased from Genechem Co. NSUN2‐knockdown lentiviruses were termed as shN‐1 and shN‐2, and control lentiviruses were termed as vector. Lentivirus used to overexpress NSUN2 was termed as N‐OE. The sequences of the NSUN2 knockdown and overexpression were as in Table S3. Lentivirus transfection after 48 h, stable cells were selected using 10 μg/mL puromycin.

### 5.5 Animals

The animal study was approved by the Institutional Animal Care and Use Committee of Xiangya Hospital Central South University (No. 2020sydw0927). BALB/c nude mice were obtained from SJA Laboratory Animal Company. 5 × 10^6^ vector‐ or shN‐KHM‐5 M cells were subcutaneously injected into female 5‐week‐old BALB/c nude mice in random groups. The tumour volumes (*V*) were calculated by the formula *V* = 1.52 × *x* × *y* ^2^, in which length (*x*) and width (*y*) of the tumours were measured at indicated time points by using a caliper. At the endpoint, mice were sacrificed. Lung metastasis mice models were intracardiac‐injected with NSUN2 knockdown and control KHM‐5 M ATC cells. Tumour tissues were collected and the weight of the tumours was measured. Mice bearing KHM‐5 M xenografts (≥100 mm^3^) were intraperitoneally injected with 4 mg/kg doxorubicin HCl^95^ or 6 mg/kg cisplatin^96^, ^97^ each 3 days with tumour volume measured. 0.9% saline injection was performed as control. The tumours were harvested at the 15^th^ day after the first drug treatment. Cisplatin (#15663‐27‐1) and doxorubicin HCl (#25316‐40‐9) were all from Selleck Chemicals.

### 5.6 IHC and IF staining

The IHC staining was performed according to the protocols as previously described.^94^ The primary antibodies (1:200−1:300 dilution) in Table S4 were used for detection of protein expression. The average optical density (AOD) was measured by ImageJ 1.53e under the same IHS range. For IF staining, cells were washed with PBS and fixed using 4% paraformaldehyde for 10 min, then permeabilised with 0.25% TritonX‐100 for 10 min, followed by blocking with 3% BSA for 30 min and incubation with primary antibodies (1:200−1:300 dilution) at 4°C overnight. The next day, used DyLight 488‐SABC Kit (Boster Biological Technology Co.) to visualise. The IF images were observed under a fluorescence microscope (Leica).

### 5.7 Western blotting

Briefly, total cellular proteins were obtained using RIPA lysis buffer (Solarbio), separated in the 10% SDS‐PAGE gel and transferred to PVDF membranes (Millipore). The membranes were incubated with the primary antibodies at 4°C overnight after blocking with 5% BSA for 30 min. The primary antibodies (1:1000 dilution) used were listed in Table S4. The next day, after incubation with specific secondary antibodies at 25°C for about 2 h, the membranes were finally visualised using ECL Chemiluminescent Substrate Reagent Kit (Invitrogen) and Amersham™ ImageQuant 800 system. GAPDH and β‐tubulin were used as internal control.

### 5.8 Quantitative reverse‐transcription polymerase chain reaction

Total RNA was extracted from the cells and tissues using Trizol reagent (AG21101) following the manufacturer's instructions. Reverse transcription was performed following instruction of Evo M‐MLV RT Mix Kit (AG 11728). After reverse transcription, the cDNAs were 1:20 diluted. And then the SYBR Green Premix Pro Taq HS qPCR Kit (Rox Plus) (AG 11718) was used in PCR reaction with three independent repeats. The reactions were performed with an Applied Biosystems QuantStudio™ 5 Real‐Time PCR Instrument (Thermo Fisher; A28134). GAPDH was used as internal control. Primers were listed in Table S2.

### 5.9 Cell proliferation, migration and invasion assays

2.0–3.0 × 10^3^ cells were separately seeded into 96‐well plates and incubated continuously at 37°C for 5 days for proliferation assay. Cell viabilities was measured using Counting Kit‐8 (CCK‐8; Beyotime). For colony‐formation assay, 300−500 cells were planted in six‐well plates and cultured with complete medium for 2 weeks. Then, cells were fixed with 4% paraformaldehyde and stained with 0.5% crystal violet, the colony numbers were counted ImageJ (version 1.53e). EdU cell proliferation assay was performed using an EdU assay kit (Cell Light EdU DNA imaging Kit; RiboBio) according to the manufacturer's instruction. 4 × 10^4^ cells were added into the upper chamber with 300 μL FBS‐free medium coated with or without Matrigel (1 mg/mL) for the transwell migration and invasion assay (24‐well, 8‐mm pore size; Corning Costar). And medium with 20% FBS was seeded into the bottom chambers. After incubating for 24 or 48 h and fixation with 4% paraformaldehyde, cells were stained with 0.5% crystal violet. Imaging was taken by inverted microscope Leica DMi8.

### 5.10 Cell cycle assay

Cells were resuspended in 500 μL propidium iodide (PI)/RNase staining buffer (BD Biosciences) after fixation with 75% PBS‐diluted ethanol overnight and incubated for 15 min at room temperature. The cell cycle was measured by flow cytometry (BD Biosciences).

### 5.11 Anti‐cancer drugs inhibition assay

Inhibitory concentration (IC50) was used to assess inhibitory effects of cisplatin or doxorubicin HCl. The experiments were performed in 96‐well plates with 3000.00 cells/100 μL 1640 RPMI complete medium including 10 % FBS. After 24 h‐treatment with eight increasing concentrations of cisplatin or doxorubicin HCl, cell viability was assessed by the optical density at 450 nm measured in a microplate reader (NanoQuant infinite M200 PRO), after adding 10 μL of CCK‐8 solution with three replicates to each well followed by incubation for 2 h at 37°C. Survival percentage at each concentration was plotted to estimate maximum half‐inhibitory concentration using GraphPad Prism (Version 9.0.0 [121]). Time‐dependent plots were plotted over time under two concentrations (higher or lower compared with IC50) of cisplatin or doxorubicin HCl.

### 5.12 tRNA bisulphite sequencing

Cells were placed on ice and treated as indicated in RNA extraction. Total RNA was resuspended in RNase‐free water. CloudSeq Inc. provided tRNA bisulphite sequencing service (Supplementary Material).

### 5.13 Puromycin intake assay

Cells were treated as indicated. Puromycin immunodetection was used for surface sensing of translation.^35^ NSUN2 was knocked down successfully by siRNAs (Table S2) targeting the 3′‐UTR of NSUN2. Cells were subjected to protein isolation and immunoblotting after incubation with medium containing 1 μM puromycin for 20 min at 37°C and 5% CO_2_ (= pulse). Anti‐puromycin antibody (#MABE343; Millipore; 1:25,000 dilution) was primary antibody in detection of neopeptide‐chain synthesis.

### 5.14 Codon usage bias analysis

CodonW 1.4.2 software (http://codonw.sourceforge.net/) was used to conduct the codon usage bias of selected protein‐coding sequence. The following indicators were used: GC content in the third digit of the synonymous codon (GC_3s_), ENC and^98^ RSCU^99^:

$$\mathrm{RSCU}\;=\;\frac{a_{km}}{\frac{1}{n_{k}}\sum_{m\;=\;1}^{n_{k}}a_{km}}$$

The amino acid under analysis has *n_k_* kinds of synonymous codons, and $a_{km}\;$stands for the observed number of the *k*‐th codon for the *m*‐th amino acid.

To evaluate the evolutionary pressure for a certain gene,^100^ relationship between ENC values and GC_3s_ was plotted and compared with ENC^expected^:

$${\mathrm{ENC}}^{\mathrm{expected}}=\;2+GC_{3s}+\frac{29}{{\mathrm{GC}}_{3s}^{2}+{\left(1-GC_{3s}\right)}^{2}}$$

To analyse the codon usage patterns in representative genes, COA based on RSCU values was used in our study.^101^ The high correlation with the codon usage pattern was represented by top dimensional vectors.

### 5.15 tRNA quantification and charging level assay

Total RNA was harvested as mentioned before. The protocol to tRNA charging level assay was adapted from Rizzino et al.^102^ with some modifications. Total RNA was resuspended in reaction buffer (10 mM acetate with 1 mM EDTA). Sodium periodate was from MACKLIN (#S817518‐25 g). Each sample was divided into two. Five micrograms was treated with either 10 mM sodium periodate (“oxidised group”) or sodium chloride with equal concentration (“non‐oxidised group”) followed by incubation for 20 min in the dark at room temperature. After using 10% glucose to quench reactions for 15 min at room temperature and being precipitated with ethanol, added yeast tRNA Phe (R4018; Sigma–Aldrich) into each sample as internal control. RNA was incubated in 50 mM Tris‐HCl buffer (pH = 9) for 50 min at 37°C to deaminoacylate, and reactions were quenched with acetate buffer.^103^ All samples were reprecipitated and resuspended in RNase‐free water. Then rtStar™ tRNA Pretreatment Kit (#AS‐FS‐004; Arraystar) was used to demethylate. Two micrograms of RNA harvested was used to perform qRT‐PCR using miRNA 1st Strand cDNA Synthesis Kit (#AG11717) and SYBR Green Premix Pro Taq HS qPCR Kit II (Rox Plus) (#AG11719) in Applied Biosystems QuantStudioTM 5 Real‐Time PCR Instrument (Thermo Fisher; A28134). U6 was used as an internal control.

### 5.16 High‐frequency codon reporter assay

Renilla luciferase is connected in‐frame to Firefly luciferase by an 18 bp sequence (6X‐TTG: 5′‐TTGTTGTTGTTGTTGTTG‐3′; 6X‐CTG: 5′‐CTGCTGCTGCTGCTGCTG‐3′) using CV094 vector in dual‐luciferase reporter system synthesised by Genechem Co. After transfection, cells were grown in 12‐well plate for 72 h. The clarified supernatant of cell lysis was extracted by lysis buffer and centrifugation. Luminescence reactions were performed using Dual Luciferase Reporter Assay Kit (Vazyme). Fluorescent signal data were measured by a Victor Plate Reader (PerkinElmer; EnVision® 2105).

### 5.17 tRNA sequencing

tRNA sequencing service and subsequent statistical analysis were provided by CloudSeq Inc. (Supplementary Material).

### 5.18 Ribosome profiling

Cells were pre‐treated with CHX at 100 mg/mL for 10 min before lysis and collection. Cell pellet was collected by centrifugation and then frozen in liquid nitrogen for >1 h for later use. CloudSeq Inc. provided ribosome profiling service (Supplementary Material) referring to the method published in *Nature protocol*.^104^

$$\mathrm{Translation}\;\mathrm{efficiency}\;=\;\frac{\mathrm{Nomalised}\;\mathrm{translation}\;\mathrm{level}\;}{\mathrm{Nomalised}\;\mathrm{mRNA}\;\mathrm{level}}$$

### 5.19 mRNA profiling

mRNA profiling service was provided by BGI Genomics.

### 5.20 LC–MS‐based tRNA modification analysis

The service was provided by Aksomics Co. (Supplementary Material). LC–MS data were acquired using Agilent Qualitative Analysis software. Multi‐reaction monitoring peaks of modified nucleoside were normalised to quantity of purified tRNA.

### 5.21 Statistical analysis

All statistical analysis was conducted using GraphPad Prism software and R (version 3.6.3). Data (outliers excluded) were presented as the mean ± standard deviation or standard error of mean. According to the characteristics of data format (homogeneity of variance or normality), appropriate statistical methods were selected for statistics, and the ggplot2 package was used for data visualisation. Correlation analysis was conducted by Spearman or Pearson method. If not otherwise indicated, ANOVA and *t*‐test were used. Survival curves were calculated using Kaplan–Meier method and log‐rank test. All statistical tests were two‐sided. *p < .05, **p < .01 and ***p < .001 were considered statistically significant.

## AUTHORS CONTRIBUTIONS

P.L., W.W., and X.L. designed the research. P.L., R.Z. and W.W. performed the experiments. P.L. analyzed data. P.L., W.W. and R.Z. wrote the manuscript. P.L., W.W. and Y.D. collected and analyzed clinical data. All authors have edited and approved the manuscript.

## CONFLICT OF INTEREST STATEMENT

The authors declare no conflict of interest.

## CONSENT FOR PUBLICATION

Not applicable.

## ETHICS APPROVAL AND CONSENT TO PARTICIPATE

This research is approved by Xiangya Hospital's Protection of Human Subjects Committee (No. 202004192) with informed consent from patients.

## Supporting information

Supporting information

Supporting information

Supporting information

Supporting information

Supporting information

Supporting information

## Data Availability

The raw data that support the findings of this study are available from the corresponding author upon reasonable request.
