# Supplementary material for: The m5C methyltransferase NSUN2 promotes codon‐dependent oncogenic translation by stabilising tRNA in anaplastic thyroid cancer
Source: Clin Transl Med. 2023 Nov 20;13(11):e1466. doi: 10.1002/ctm2.1466 (PMC10659772; doi:10.1002/ctm2.1466)
Supplement: Supplementary file 1 — Supporting information [file CTM2-13-e1466-s002.docx]

**tRNA bisulfite sequencing**

Cells were treated as indicated in RNA extraction, placed on ice. Total RNA wasresuspended in RNase-free water. tRNA bisulfite sequencing service was provided by CloudSeq Inc. (Shanghai, China). Total RNA was size-selected for small RNA fraction (<200nt) with the MirVana Isolation Kit (ThermoFisher). The enriched small RNAs were de-aminoacylated in 0.1 M Tris–HCl pH 9.0 and 1 mM EDTA for 30 min at 37°C. De-aminoacylated RNA was bisulfite converted and purified using the EZ RNA methylation Kit (Zymo Research). tRNA libraries were constructed with GenSeq® Small RNA Library Prep Kit (GenSeq, Inc.) by following the manufacturer’s instructions, and then sequenced in a NovaSeq platform (Illumina, Inc., San Diego, CA, USA).

**tRNA sequencing**

tRNA sequencing service was provided by CloudSeq Inc. (Shanghai, China). Total RNA was size-selected for small RNA fraction (<200nt) with the MirVana Isolation Kit (ThermoFisher). The enriched small RNAs were de-aminoacylated in Tris–HCl pH 9.0 with 1 mM EDTA for 30 min at 37°C. tRNA libraries were constructed with GenSeq® Small RNA Library Prep Kit (GenSeq, Inc.) by following the manufacturer’s instructions. All libraries were size-selected for tRNA fraction before sequencing and then sequenced in a NovaSeq platform (Illumina, Inc., San Diego, CA, USA).

**Ribosome profiling**

Ribosome profiling service was provided by CloudSeq Inc. (Shanghai, China). Cells were pre-treated with CHX at 100 mg/ml for 10 minutes before lysis and collection. Referring to the method published in *Nature protocol*^104^, the lysate solution was first digested by ribonuclease and separated ribosome bounding RNA from free mRNA. RPFs were obtained by enrichment of ribosome-mRNA complex. After removing rRNA and purifying with PAGE gel, the target RNA fragments were obtained. Added 5’ and 3’ adaptor directly to the ends. cDNA was synthesized by reverse transcription and was purified by PAGE gel. The initial library was obtained by adding adaptors and then PCR enrichment. PAGE gel was used to screen target fragments. The constructed cDNA library sequencing was conducted by using Illumina HiSeqTM X10 platform by Gene Denovo Biotechnology Co.

**LC-MS based tRNA modification analysis**

The service was provided by Aksomics Co. (Shanghai, China). tRNA was isolated from total RNA samples using NEBNext Poly(A) tRNA Magnetic Isolation Module (NEB, E7490). Purified tRNA was quantified using Qubit RNA HS Assay kit (ThermoFisher, Q32855). tRNA was hydrolyzed to single nucleosides. Then nucleosides were dephosphorylated by enzyme mixture. Pretreated nucleosides solution was deproteinized by using Satorius 10,000-Da MWCO spin filter. Analysis of nucleoside mixtures was performed on Agilent 6460 QQQ mass spectrometer with Agilent 1260 HPLC system.LC-MS data was acquired using Agilent Qualitative Analysis software. Multi-reaction monitoring peaks of each modified nucleoside were extracted and normalized to quantity of purified tRNA.
