# Supplementary material for: The m5C methyltransferase NSUN2 promotes codon‐dependent oncogenic translation by stabilising tRNA in anaplastic thyroid cancer
Source: Clin Transl Med. 2023 Nov 20;13(11):e1466. doi: 10.1002/ctm2.1466 (PMC10659772; doi:10.1002/ctm2.1466)
Supplement: Supplementary file 2 — Supporting information [file CTM2-13-e1466-s003.pptx]

## Slide 1
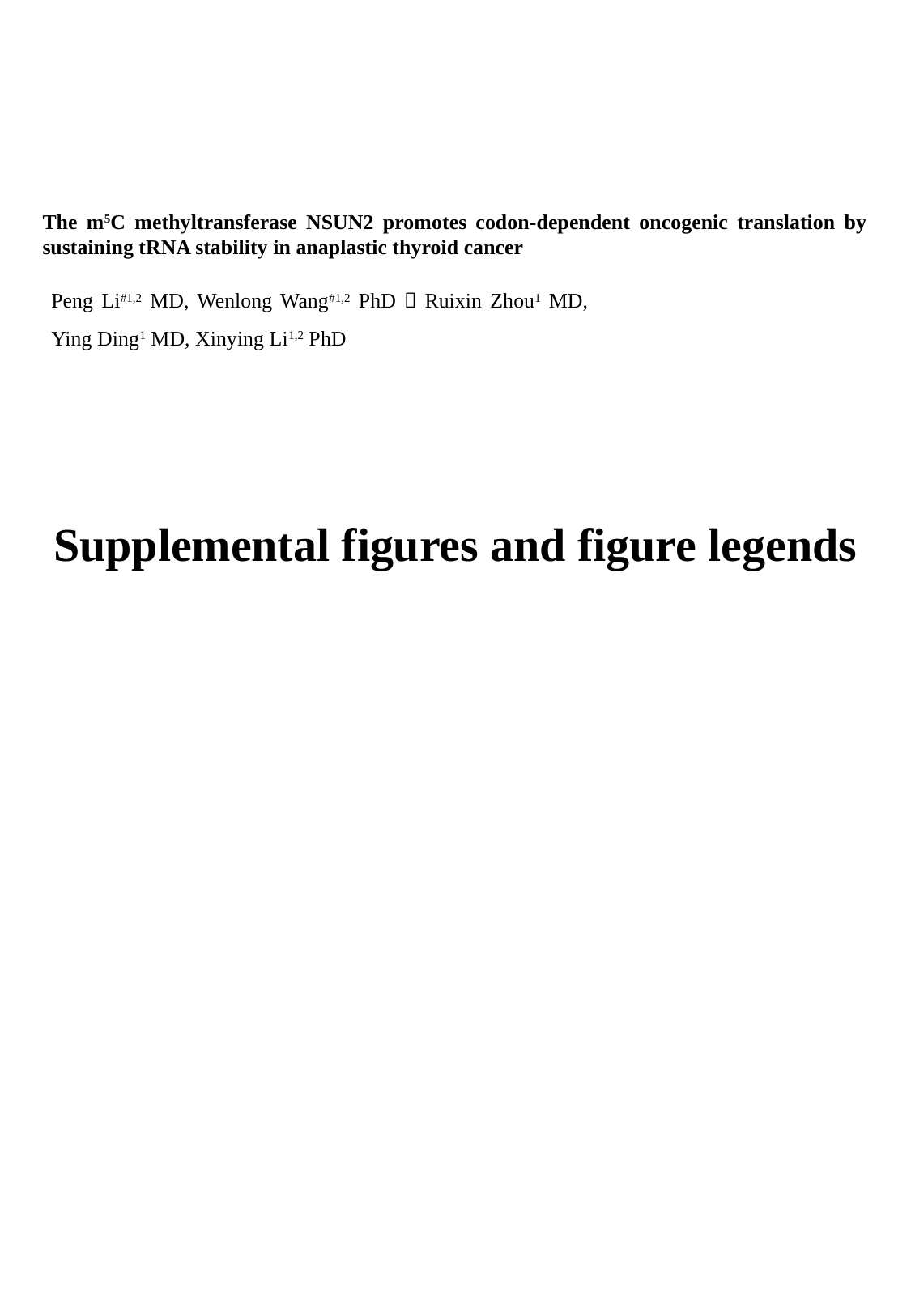

The m5C methyltransferase NSUN2 promotes codon-dependent oncogenic translation by sustaining tRNA stability in anaplastic thyroid cancer
Peng Li#1,2 MD, Wenlong Wang#1,2 PhD，Ruixin Zhou1 MD, Ying Ding1 MD, Xinying Li1,2 PhD
# Supplemental figures and figure legends

## Slide 2
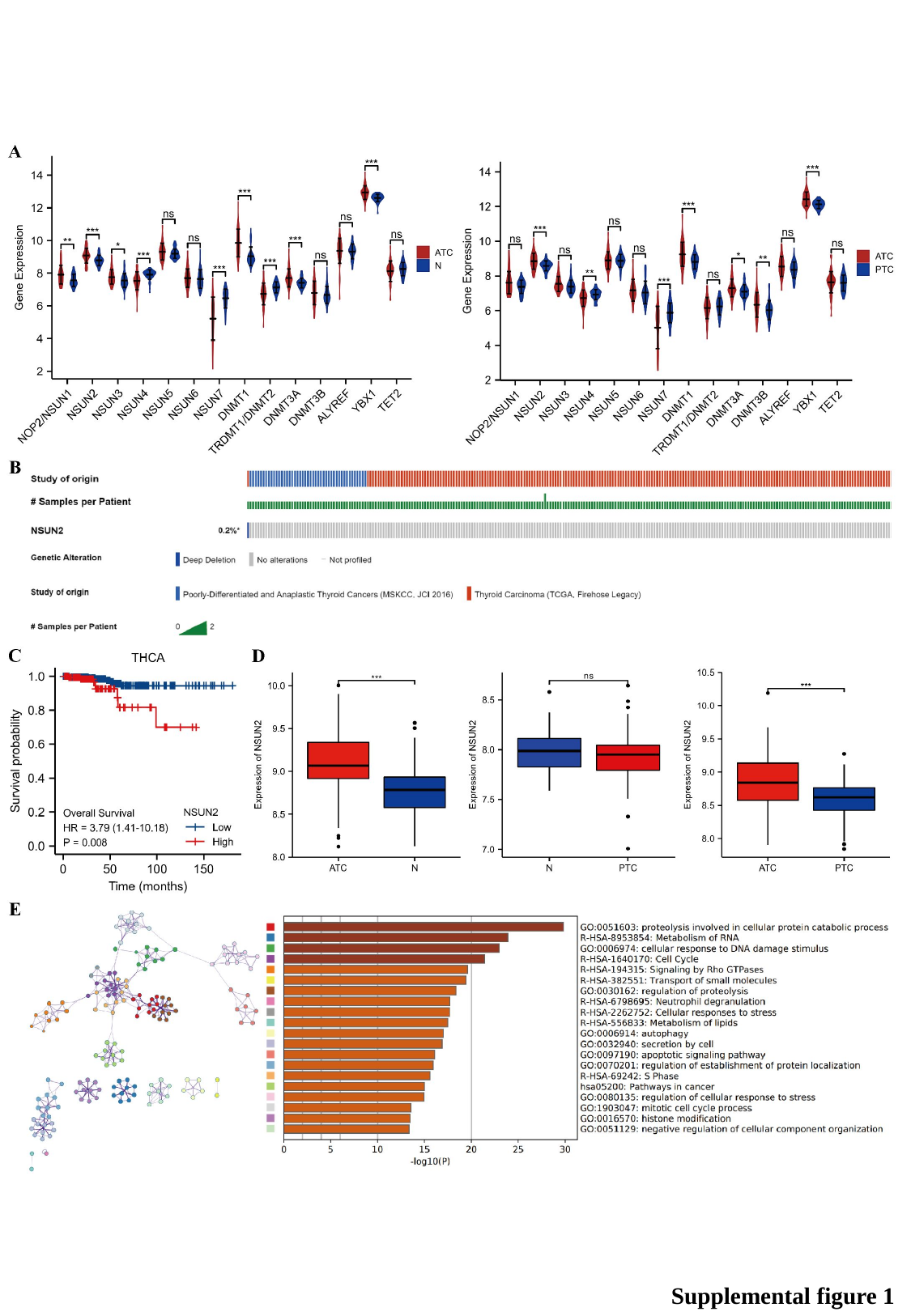

Supplemental figure 1

## Slide 3
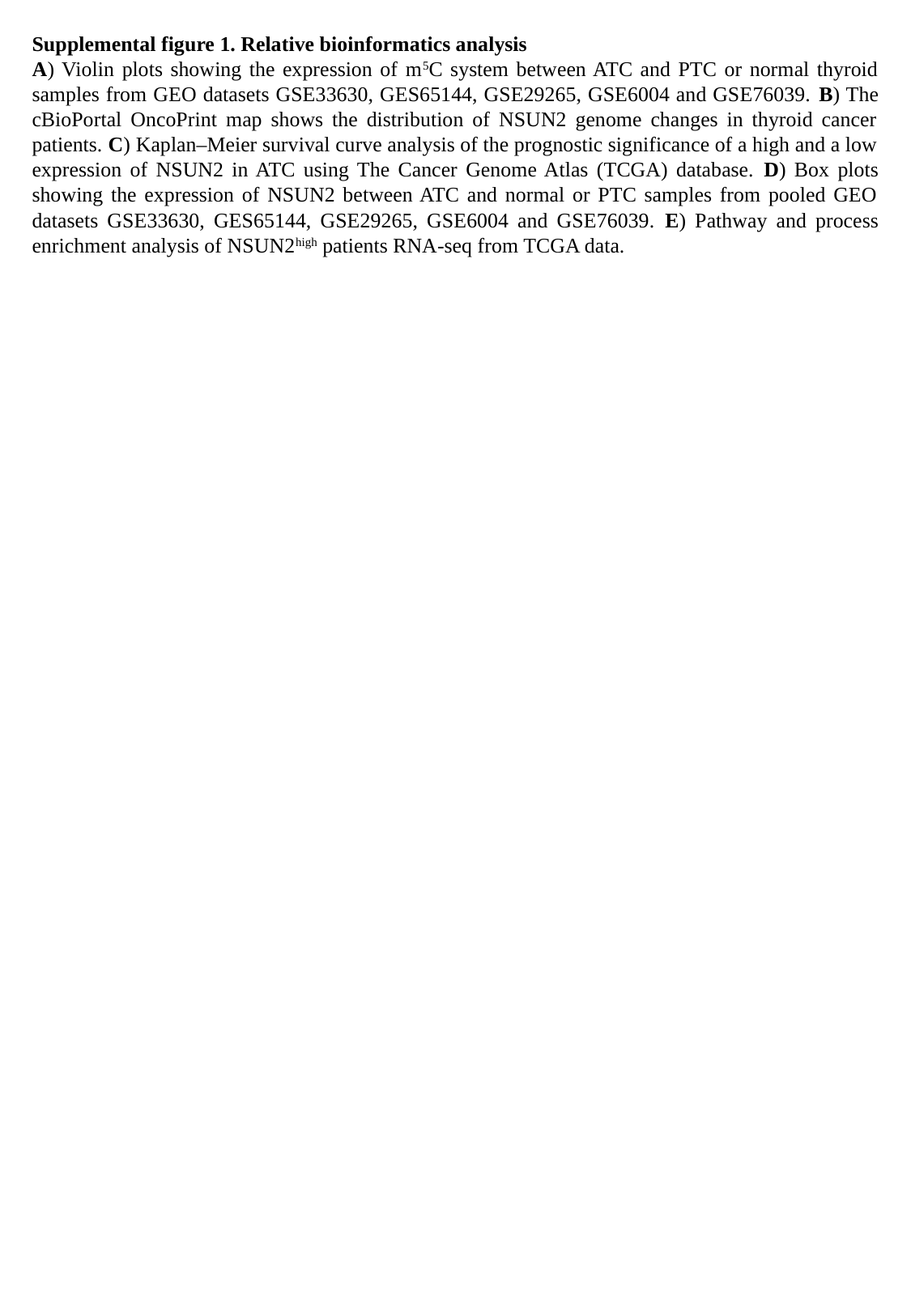

Supplemental figure 1. Relative bioinformatics analysis
A) Violin plots showing the expression of m5C system between ATC and PTC or normal thyroid samples from GEO datasets GSE33630, GES65144, GSE29265, GSE6004 and GSE76039. B) The cBioPortal OncoPrint map shows the distribution of NSUN2 genome changes in thyroid cancer patients. C) Kaplan–Meier survival curve analysis of the prognostic significance of a high and a low expression of NSUN2 in ATC using The Cancer Genome Atlas (TCGA) database. D) Box plots showing the expression of NSUN2 between ATC and normal or PTC samples from pooled GEO datasets GSE33630, GES65144, GSE29265, GSE6004 and GSE76039. E) Pathway and process enrichment analysis of NSUN2high patients RNA-seq from TCGA data.

## Slide 4
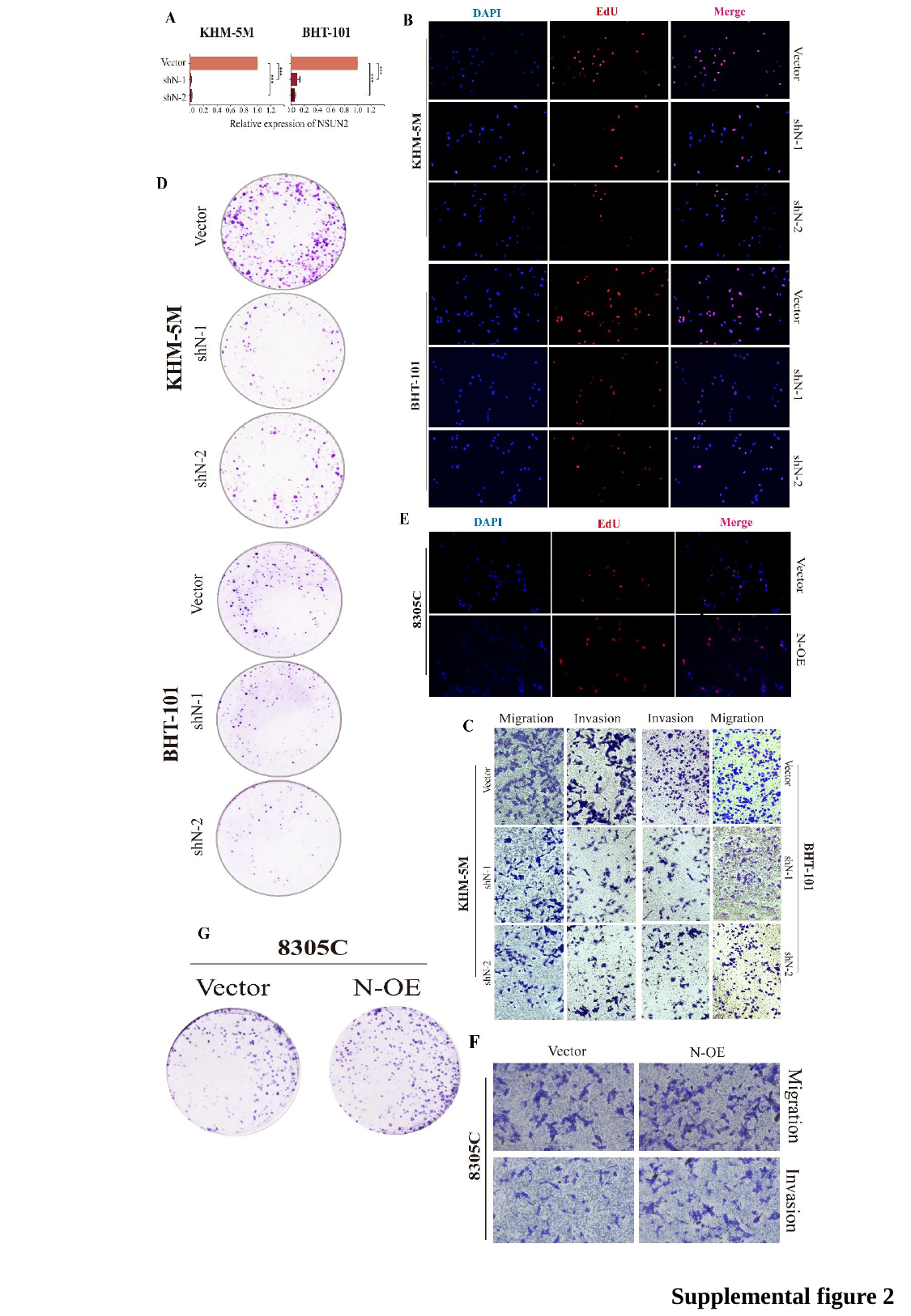

Supplemental figure 2

## Slide 5
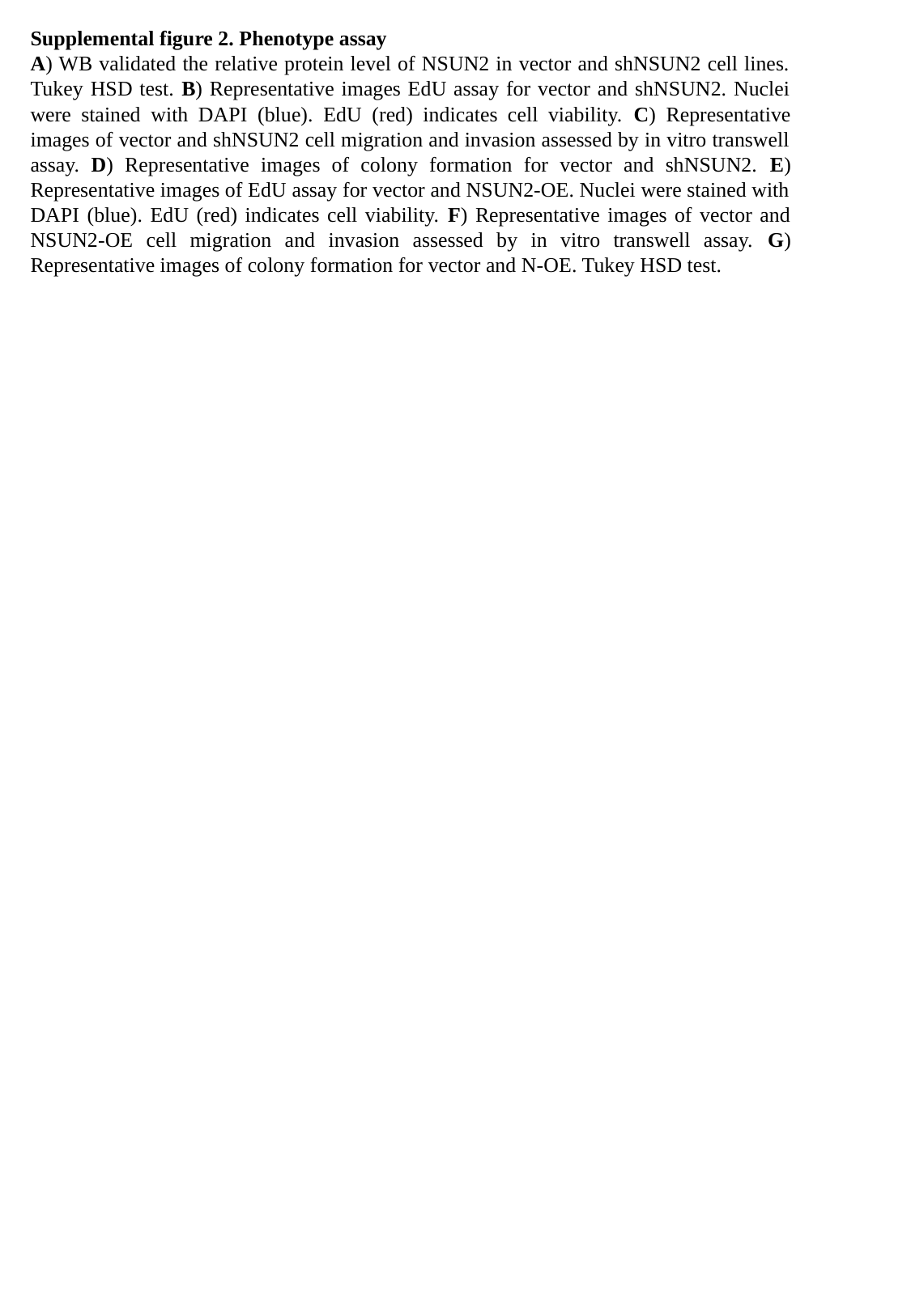

Supplemental figure 2. Phenotype assay
A) WB validated the relative protein level of NSUN2 in vector and shNSUN2 cell lines. Tukey HSD test. B) Representative images EdU assay for vector and shNSUN2. Nuclei were stained with DAPI (blue). EdU (red) indicates cell viability. C) Representative images of vector and shNSUN2 cell migration and invasion assessed by in vitro transwell assay. D) Representative images of colony formation for vector and shNSUN2. E) Representative images of EdU assay for vector and NSUN2-OE. Nuclei were stained with DAPI (blue). EdU (red) indicates cell viability. F) Representative images of vector and NSUN2-OE cell migration and invasion assessed by in vitro transwell assay. G) Representative images of colony formation for vector and N-OE. Tukey HSD test.

## Slide 6
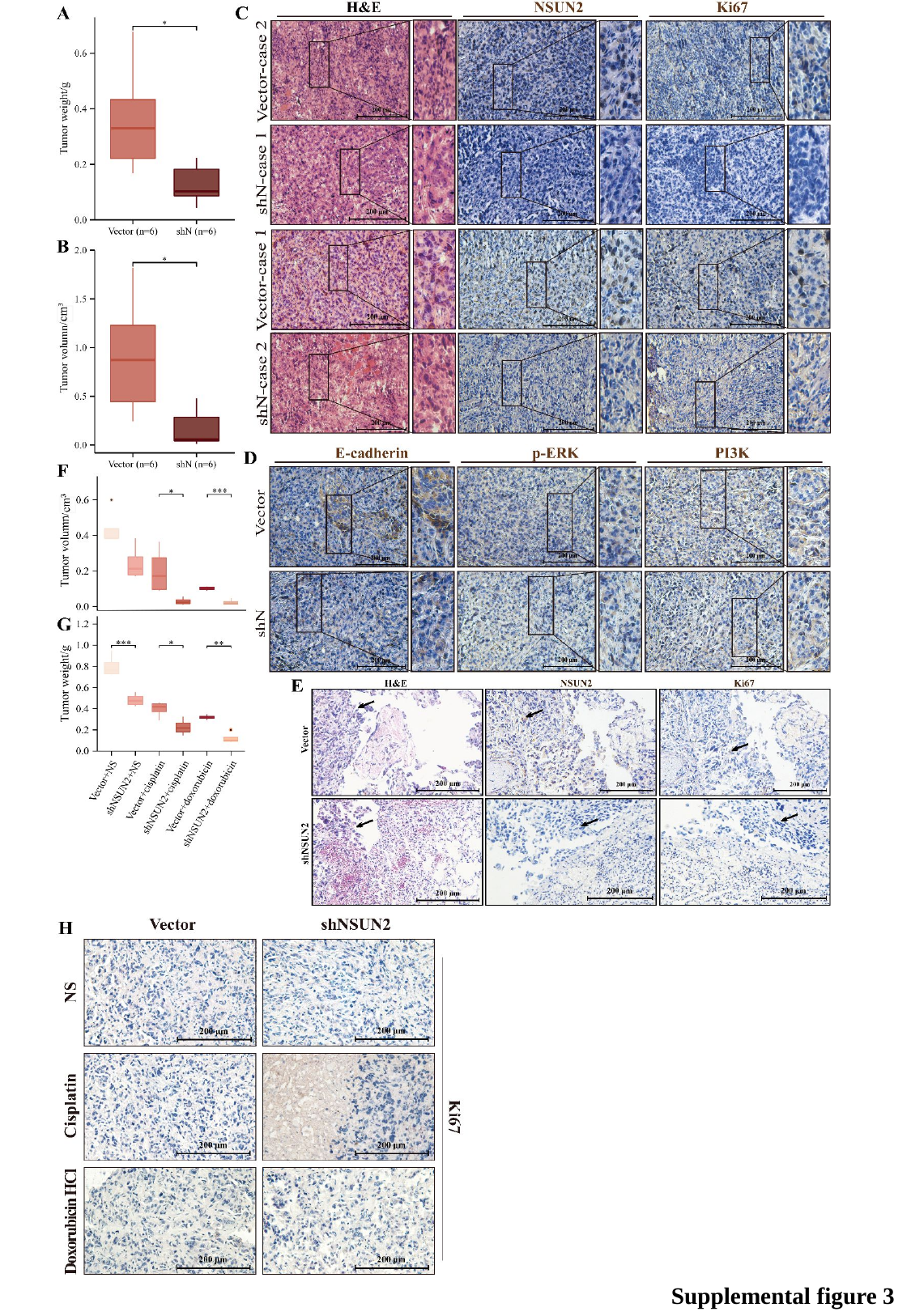

Supplemental figure 3

## Slide 7
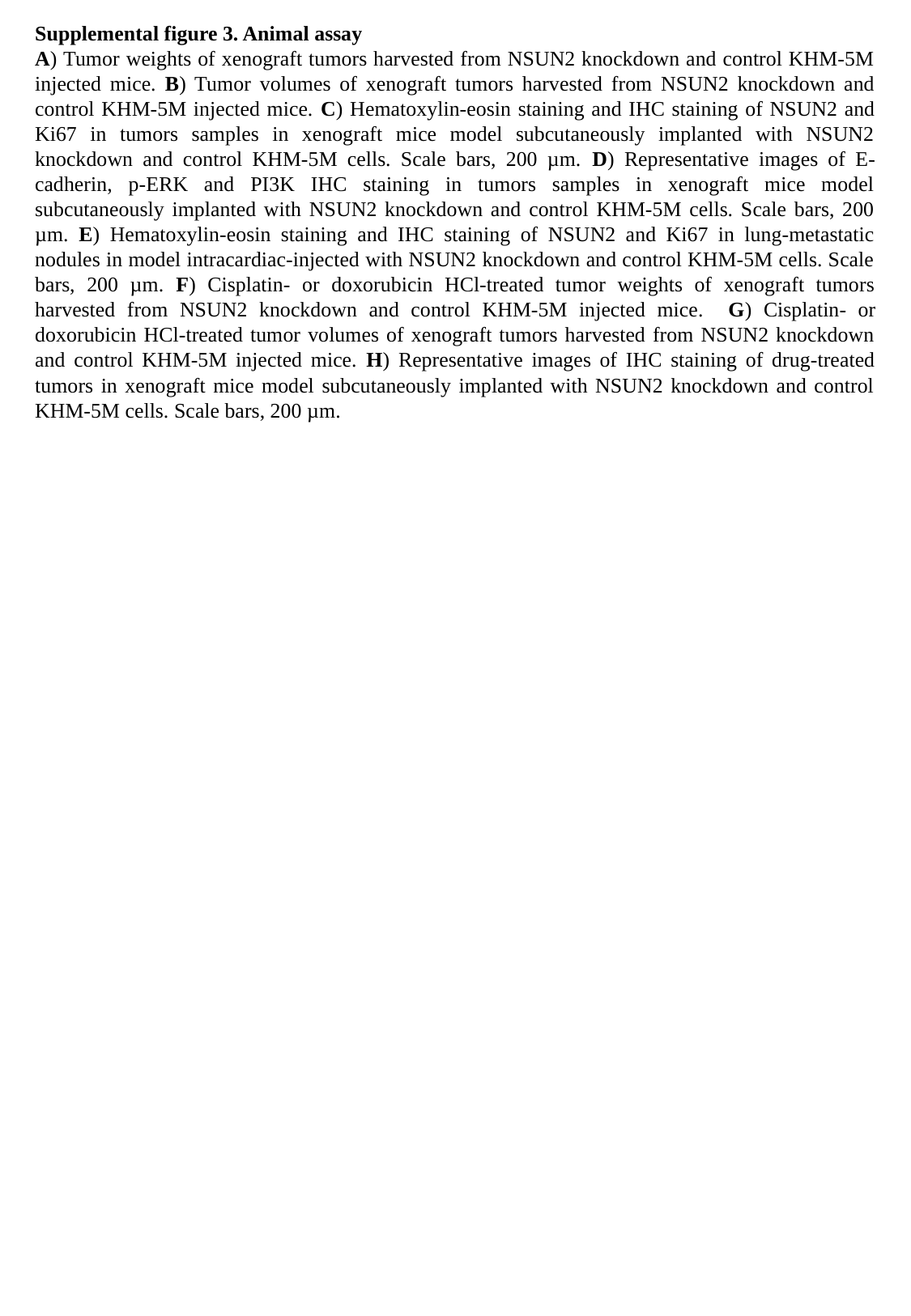

Supplemental figure 3. Animal assay
A) Tumor weights of xenograft tumors harvested from NSUN2 knockdown and control KHM-5M injected mice. B) Tumor volumes of xenograft tumors harvested from NSUN2 knockdown and control KHM-5M injected mice. C) Hematoxylin-eosin staining and IHC staining of NSUN2 and Ki67 in tumors samples in xenograft mice model subcutaneously implanted with NSUN2 knockdown and control KHM-5M cells. Scale bars, 200 µm. D) Representative images of E-cadherin, p-ERK and PI3K IHC staining in tumors samples in xenograft mice model subcutaneously implanted with NSUN2 knockdown and control KHM-5M cells. Scale bars, 200 µm. E) Hematoxylin-eosin staining and IHC staining of NSUN2 and Ki67 in lung-metastatic nodules in model intracardiac-injected with NSUN2 knockdown and control KHM-5M cells. Scale bars, 200 µm. F) Cisplatin- or doxorubicin HCl-treated tumor weights of xenograft tumors harvested from NSUN2 knockdown and control KHM-5M injected mice. G) Cisplatin- or doxorubicin HCl-treated tumor volumes of xenograft tumors harvested from NSUN2 knockdown and control KHM-5M injected mice. H) Representative images of IHC staining of drug-treated tumors in xenograft mice model subcutaneously implanted with NSUN2 knockdown and control KHM-5M cells. Scale bars, 200 µm.

## Slide 8
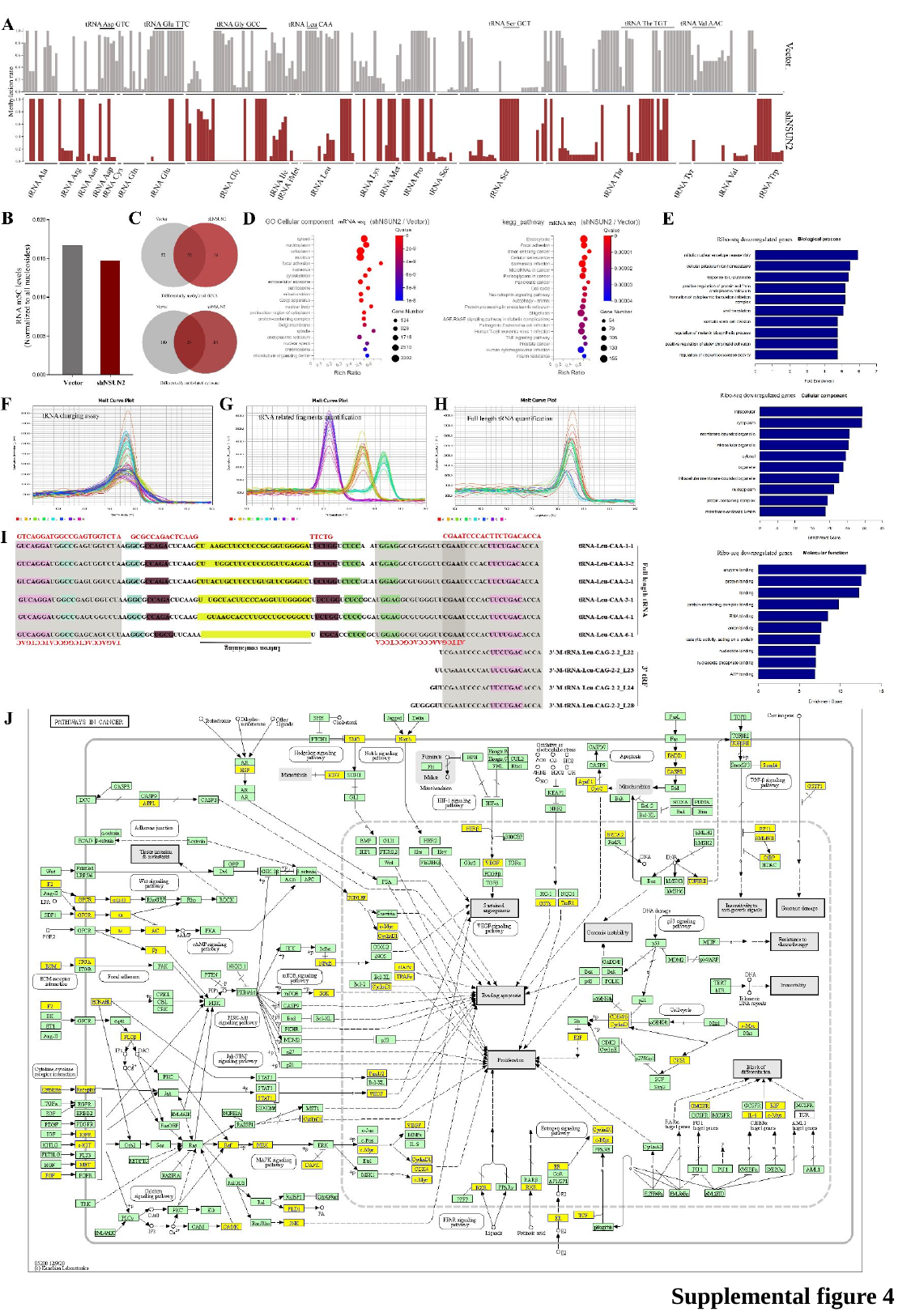

Supplemental figure 4

## Slide 9
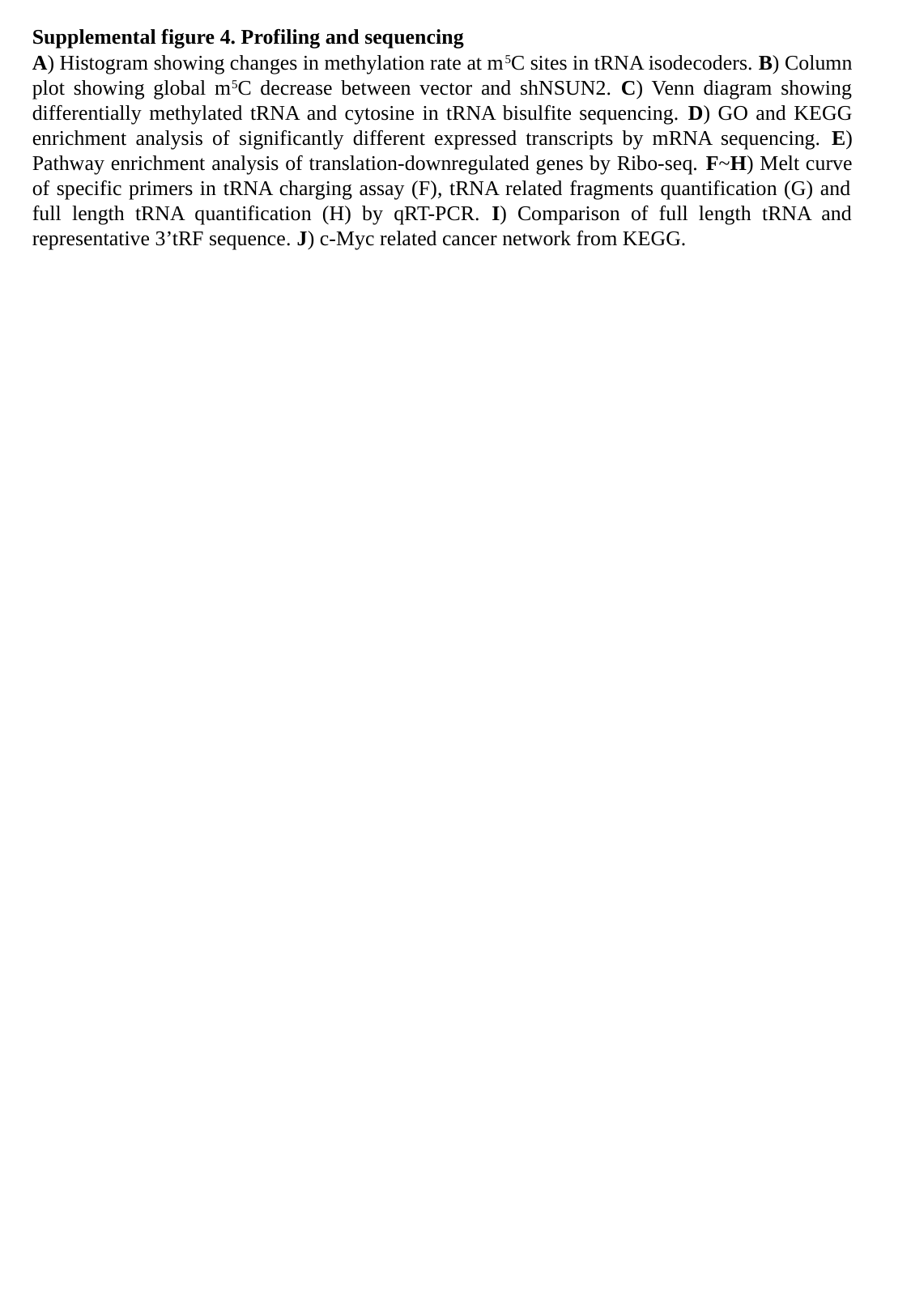

Supplemental figure 4. Profiling and sequencing
A) Histogram showing changes in methylation rate at m5C sites in tRNA isodecoders. B) Column plot showing global m5C decrease between vector and shNSUN2. C) Venn diagram showing differentially methylated tRNA and cytosine in tRNA bisulfite sequencing. D) GO and KEGG enrichment analysis of significantly different expressed transcripts by mRNA sequencing. E) Pathway enrichment analysis of translation-downregulated genes by Ribo-seq. F~H) Melt curve of specific primers in tRNA charging assay (F), tRNA related fragments quantification (G) and full length tRNA quantification (H) by qRT-PCR. I) Comparison of full length tRNA and representative 3’tRF sequence. J) c-Myc related cancer network from KEGG.

## Slide 10
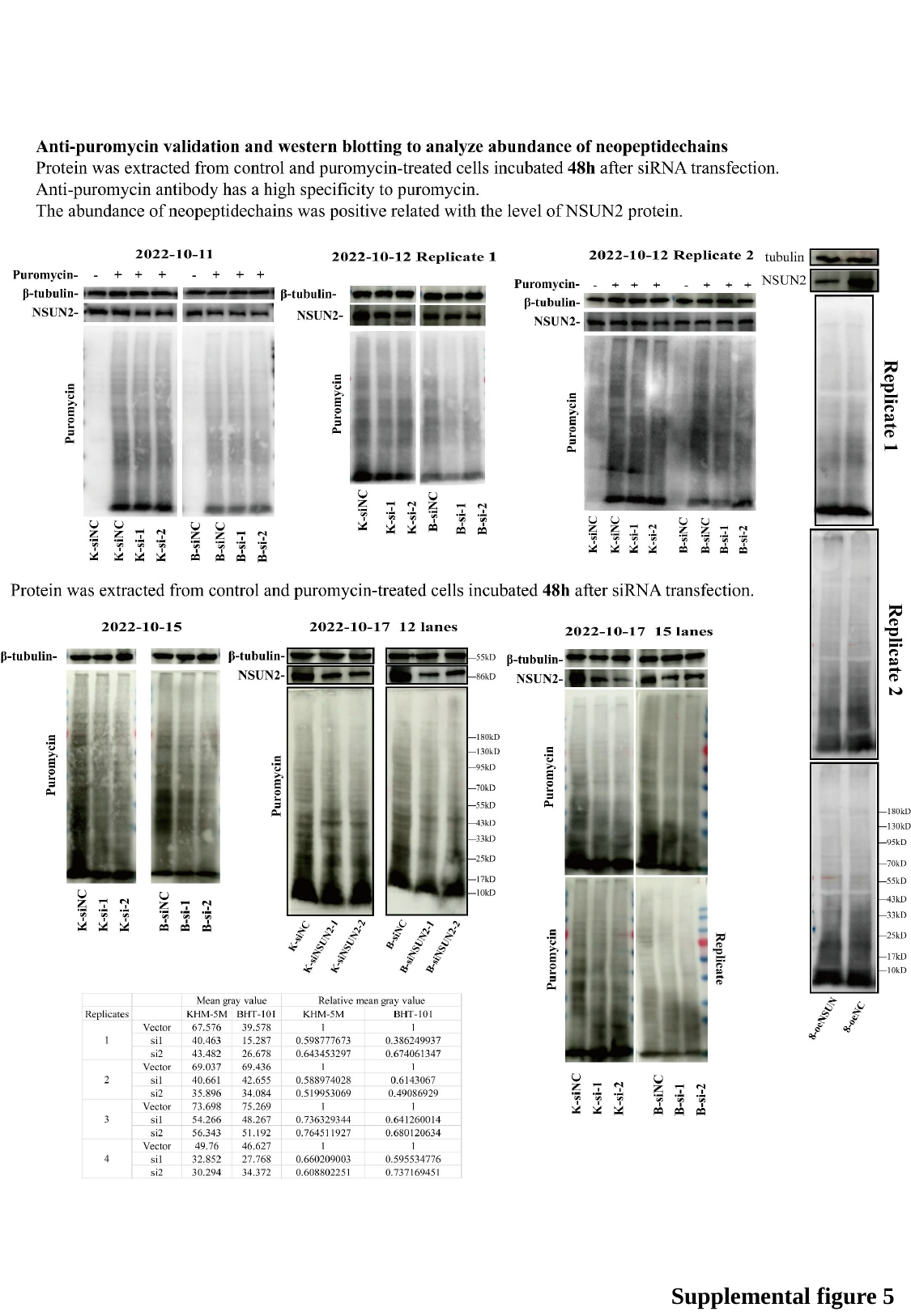

Supplemental figure 5

## Slide 11
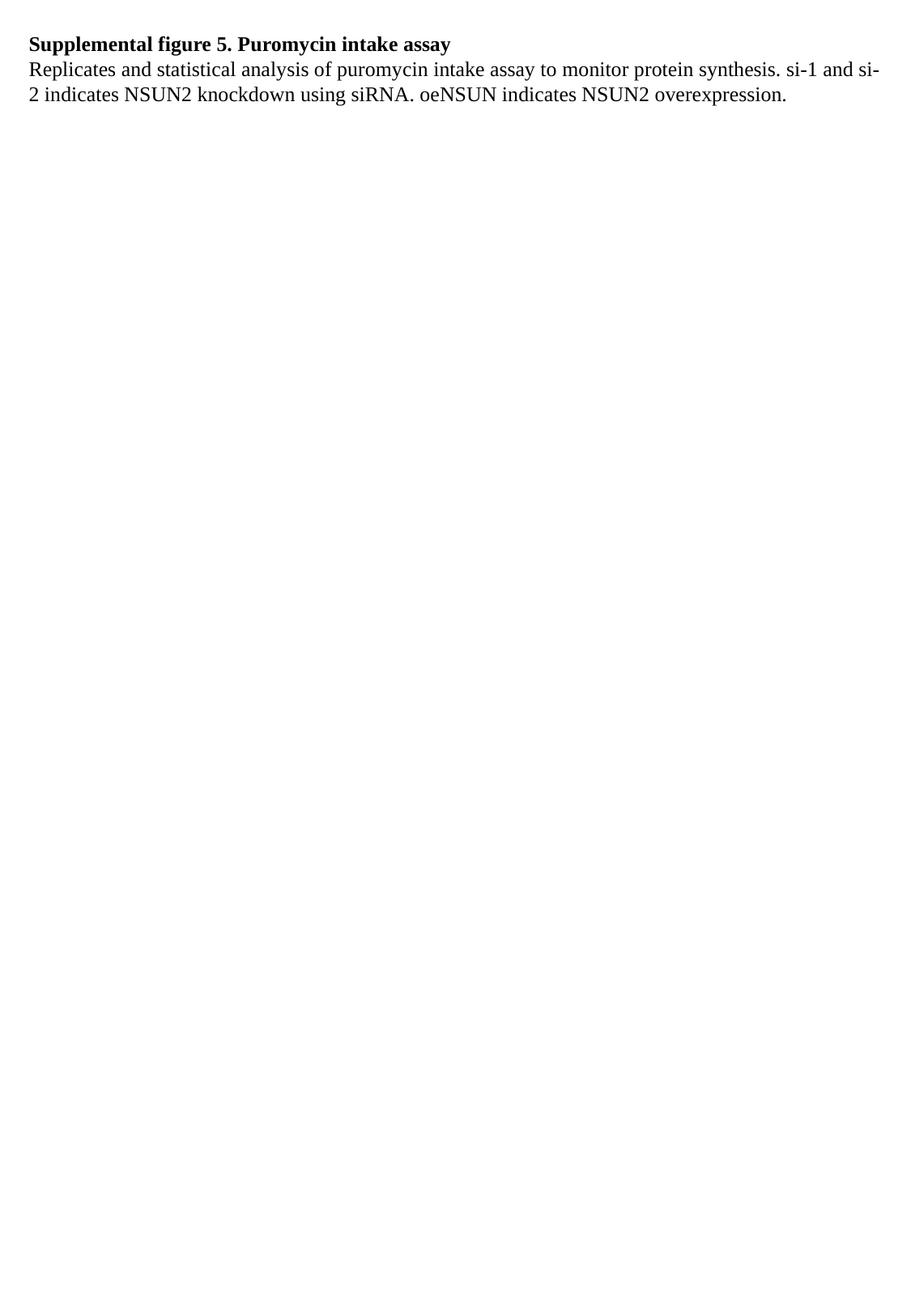

Supplemental figure 5. Puromycin intake assay
Replicates and statistical analysis of puromycin intake assay to monitor protein synthesis. si-1 and si-2 indicates NSUN2 knockdown using siRNA. oeNSUN indicates NSUN2 overexpression.
