# Supplementary material for: The m5C methyltransferase NSUN2 promotes codon‐dependent oncogenic translation by stabilising tRNA in anaplastic thyroid cancer
Source: Clin Transl Med. 2023 Nov 20;13(11):e1466. doi: 10.1002/ctm2.1466 (PMC10659772; doi:10.1002/ctm2.1466)
Supplement: Supplementary file 3 — Supporting information [file CTM2-13-e1466-s004.docx]

| **Supplementary Table 1 IC50 of NSUN2 knockdown and overexpression cells to cisplatin and doxorubicin HCl** | | | | | | |
| --- | --- | --- | --- | --- | --- | --- |
|  |  |  | Cisplatin | | Doxorubicin HCl | |
|  | Replicates |  | IC50 | 95%CI | IC50 | 95%CI |
| KHM-5M | 1 | Vector | 23.08 | 16.72-31.95 | 4.90 | 3.701-6.538 |
|  |  | shN-1 | 19.14 | 14.07-25.99 | 3.55 | 2.793-4.516 |
|  |  | shN-2 | 17.43 | 11.96-25.32 | 3.37 | 2.625-4.337 |
|  | 2 | Vector | 22.74 | 16.99-30.52 | 5.03 | 3.874-6.572 |
|  |  | shN-1 | 19.81 | 13.18-29.78 | 3.01 | 2.444-3.714 |
|  |  | shN-2 | 17.79 | 12.51-25.22 | 3.22 | 2.755-3.776 |
|  | 3 | Vector | 24.34 | 15.78-37.34 | 4.46 | 3.620-5.496 |
|  |  | shN-1 | 17.11 | 11.82-24.64 | 3.09 | 2.295-4.974 |
|  |  | shN-2 | 15.32 | 11.40-20.48 | 2.67 | 2.012-4.636 |
| BHT-101 | 1 | Vector | 14.00 | 9.744-19.98 | 3.47 | 2.506-4.800 |
|  |  | shN-1 | 10.81 | 7.990-14.470 | 2.02 | 1.742-2.466 |
|  |  | shN-2 | 9.50 | 7.131-12.51 | 2.19 | 1.587-3.027 |
|  | 2 | Vector | 12.62 | 8.864-17.81 | 3.69 | 2.682-5.116 |
|  |  | shN-1 | 9.93 | 7.707-12.69 | 2.55 | 1.934-3.338 |
|  |  | shN-2 | 8.68 | 6.600-11.28 | 2.04 | 1.808-2.415 |
|  | 3 | Vector | 13.25 | 9.132-19.25 | 3.22 | 2.505-5.262 |
|  |  | shN-1 | 8.60 | 6.546-11.15 | 1.86 | 1.566-2.365 |
|  |  | shN-2 | 8.33 | 6.513-10.54 | 2.21 | 1.665-2.937 |
| 8305C | 1 | Vector | 16.00 | 13.90-18.46 | 2.30 | 1.280-4.266 |
|  |  | N-OE | 17.35 | 15.04-19.84 | 2.97 | 1.997-4.493 |
|  | 2 | Vector | 15.03 | 13.07-17.32 | 2.33 | 1.499-3.804 |
|  |  | N-OE | 18.72 | 16.91-20.75 | 2.91 | 1.840-4.687 |
|  | 3 | Vector | 15.97 | 13.00-19.72 | 2.23 | 1.120-4.698 |
|  |  | N-OE | 19.37 | 17.60-21.36 | 2.99 | 1.966-4.598 |
