# Supplementary material for: The m5C methyltransferase NSUN2 promotes codon‐dependent oncogenic translation by stabilising tRNA in anaplastic thyroid cancer
Source: Clin Transl Med. 2023 Nov 20;13(11):e1466. doi: 10.1002/ctm2.1466 (PMC10659772; doi:10.1002/ctm2.1466)
Supplement: Supplementary file 4 — Supporting information [file CTM2-13-e1466-s006.docx]

| **Supplemental Table 2 Primers and siRNAs** | | |
| --- | --- | --- |
| **Primers** | | |
| **NCBI gene symbol/Name** | **Forward (5'→3')** | **Reverse (3'→5')** |
| **NSUN2** | GAACTTGCCTGGCACACAAAT | TGCTAACAGCTTCTTGACGACTA |
| **GAPDH** | GGAGCGAGATCCCTCCAAAAT | GGCTGTTGTCATACTTCTCATGG |
| **c-Myc** | GTCAAGAGGCGAACACACAAC | TTGGACGGACAGGATGTATGC |
| **TRAF2** | GCTCATGCTGACCGAATGTC | GCCGTCACAAGTTAAGGGGAA |
| **BCL2** | GGTGGGGTCATGTGTGTGG | CGGTTCAGGTACTCAGTCATCC |
| **RAB31** | GGGGTTGGGAAATCAAGCATC | GCCAATGAATGAAACCGTTCCT |
| **JUNB** | ACAAACTCCTGAAACCGAGCC | CGAGCCCTGACCAGAAAAGTA |
| **VEGFA** | AGGGCAGAATCATCACGAAGT | AGGGTCTCGATTGGATGGCA |
| **VEGFB** | GAGATGTCCCTGGAAGAACACA | GAGTGGGATGGGTGATGTCAG |
| **VEGFC** | ATGTGTGTCCGTCTACAGATGT | GGAAGTGTGATTGGCAAAACTGA |
| **Charging assay-yaest-tRNA Phe-GAA** | GCGGACTTAGCTCAGTTGGGAGAG | - (polyA 3' specific primer) |
| **Charging assay-Leu-CAA** | GCGCCAGACTCAAGTTCTG | - (polyA 3' specific primer) |
| **Charging assay-Leu-CAG** | TTCAGGTCGCAGTCTCCC | - (polyA 3' specific primer) |
| **tRNA Leu-CAA** | GCGCCAGACTCAAGTTCTG | TAGACCACTCGGCCATCCTGAC |
| **tRNA Leu-CAA 3' related fragments** | CGAATCCCACTTCTGACACCA | - (polyA 3' specific primer) |
| **Precursor and mature tRNA Leu-CAA** | GTCAGGATGGCCGAGTGGTCTA | ATTCGAACCCACGCCTCCA |
| **Mature tRNA Leu-CAA** | GTCAGGATGGCCGAGTGGTCTA | TGGTGTCAGAAGTGGGATTCG |
| **U6** | CTCGCTTCGGCAGCACA | AACGCTTCACGAATTTGCGT |
| **siRNA** | | |
| **Name** | **Squence (5'-3')** | |
| **hNSUN2 si1 sense** | CAGUGGAAGGUAAUGACGAAATT | |
| **hNSUN2 si1 anti-sense** | UUUCGUCAUUACCUUCCACUGTT | |
| **hNSUN2 si2 sense** | UGCAGUGUCCCAUCGUCUUAUTT | |
| **hNSUN2 si2 anti-sense** | AUAAGACGAUGGGACACUGCATT | |
| **hMYC si2 sense** | CGAGCUAAAACGGAGCUUUTT | |
| **hMYC si2 anti-sense** | AAAGCUCCGUUUUAGCUCGTT | |
