# Supplementary material for: The m5C methyltransferase NSUN2 promotes codon‐dependent oncogenic translation by stabilising tRNA in anaplastic thyroid cancer
Source: Clin Transl Med. 2023 Nov 20;13(11):e1466. doi: 10.1002/ctm2.1466 (PMC10659772; doi:10.1002/ctm2.1466)
Supplement: Supplementary file 5 — Supporting information [file CTM2-13-e1466-s001.docx]

| **Supplementary Table 3-shRNA and Overexpression** | | | | | | |
| --- | --- | --- | --- | --- | --- | --- |
| **Name** | | **5’** | **STEM** | **Loop** | **STEM** | **3’** |
| shN | shN1-NSUN2-RNAi(108552-1)-a | Ccgg | gaGCGATGCCTTAGGATATTA | CTCGAG | TAATATCCTAAGGCATCGCTC | TTTTTg |
|  | shN1-NSUN2-RNAi(108552-1)-b | aattcaaaaa | gaGCGATGCCTTAGGATATTA | CTCGAG | TAATATCCTAAGGCATCGCTC |  |
|  | shN2-NSUN2-RNAi(108553-1)-a | Ccgg | caGTGGAAGGTAATGACGAAA | CTCGAG | TTTCGTCATTACCTTCCACTG | TTTTTg |
|  | shN2-NSUN2-RNAi(108553-1)-b | aattcaaaaa | caGTGGAAGGTAATGACGAAA | CTCGAG | TTTCGTCATTACCTTCCACTG |  |
| N-OE/oeNSUN2 | CCGTTTTTGGCTTTTTTGTTAGACGAAGCTTGGGCTGCAGGTCGACTCTAGAGGATCCAACTTTGTGCCAACCGGTCGCCACCATGGGGCGGCGGTCGCGGGGTCGGCGGCTCCAGCAACAGCAGCGGCCGGAAGACGCGGAGGATGGCGCCGAGGGTGGTGGAAAGCGCGGCGAGGCGGGCTGGGAAGGAGGCTACCCCGAGATCGTCAAGGAGAACAAGCTGTTCGAGCACTACTACCAGGAGCTCAAGATCGTGCCCGAGGGCGAGTGGGGCCAGTTCATGGACGCTCTCAGGGAGCCGCTCCCGGCCACTTTAAGAATTACTGGTTACAAAAGCCACGCAAAAGAGATTCTCCATTGCTTAAAGAACAAATATTTTAAGGAATTGGAGGACCTGGAGGTGGACGGTCAGAAAGTTGAAGTTCCACAGCCACTGAGTTGGTATCCTGAAGAACTTGCCTGGCACACAAATTTAAGTCGAAAAATCTTGAGAAAATCGCCACACTTGGAAAAGTTTCATCAGTTTCTAGTTAGTGAAACAGAATCTGGAAATATTAGTCGTCAAGAAGCTGTTAGCATGATCCCACCACTGCTCCTCAACGTGCGGCCTCATCATAAGATCTTAGATATGTGTGCAGCACCTGGCTCAAAGACCACACAGTTAATTGAAATGCTACATGCCGACATGAATGTCCCCTTTCCAGAGGGATTTGTTATTGCGAATGATGTGGACAACAAGCGCTGCTACCTGCTCGTCCATCAAGCCAAGAGGCTGAGCAGCCCCTGCATCATGGTGGTCAACCATGATGCCTCCAGCATACCCAGGCTCCAGATAGATGTGGACGGCAGGAAAGAGATCCTCTTCTATGATCGAATTTTATGTGATGTCCCTTGCAGTGGAGACGGCACTATGAGAAAAAACATTGATGTTTGGAAAAAGTGGACCACCTTAAATAGCTTGCAGCTACATGGCTTACAGCTGCGGATTGCAACACGCGGGGCTGAACAGCTGGCTGAAGGTGGAAGGATGGTGTATTCCACGTGTTCACTAAACCCTATTGAGGATGAAGCAGTCATAGCATCTTTACTGGAAAAAAGTGAAGGTGCTTTGGAGCTTGCTGATGTGTCTAATGAACTGCCAGGGCTGAAGTGGATGCCTGGAATCACACAGTGGAAGGTAATGACGAAAGATGGGCAGTGGTTTACAGACTGGGACGCTGTTCCTCACAGCAGACACACCCAGATCCGACCTACCATGTTCCCTCCGAAGGACCCAGAAAAGCTGCAGGCCATGCACCTGGAGCGATGCCTTAGGATATTACCCCATCATCAGAATACTGGAGGGTTTTTTGTGGCAGTATTGGTGAAAAAATCTTCAATGCCGTGGAATAAACGTCAGCCAAAGCTTCAGGGTAAATCTGCAGAGACCAGAGAAAGCACACAGCTGAGCCCTGCAGATCTCACAGAAGGGAAACCCACAGATCCCTCTAAGCTGGAAAGTCCGTCATTCACAGGAACTGGTGACACAGAAATAGCTCATGCAACTGAGGATTTAGAGAATAATGGCAGTAAGAAAGATGGCGTGTGTGGTCCTCCTCCATCAAAGAAAATGAAGTTATTTGGATTTAAAGAAGATCCATTTGTATTTATTCCTGAAGATGACCCATTATTTCCACCTATTGAGAAATTTTATGCTTTGGATCCTTCATTCCCAAGGATGAATTTGTTAACTCGGACTACAGAAGGGAAGAAAAGGCAGCTCTACATGGTTTCTAAGGAGTTGCGGAATGTGCTGCTGAATAACAGTGAGAAGATGAAGGTTATTAACACGGGGATCAAAGTCTGGTGTAGAAATAACAGCGGTGAAGAGTTTGACTGTGCTTTCCGGCTGGCACAGGAGGGAATATATACATTGTATCCATTTATTAACTCAAGAATTATTACTGTATCAATGGAAGATGTTAAGATACTGTTGACCCAGGAAAATCCCTTTTTTAGAAAACTCAGCAGTGAGACCTACAGTCAAGCAAAGGACCTGGCAAAGGGAAGCATCGTGCTGAAGTATGAACCAGATTCTGCGAATCCAGACGCTCTGCAGTGTCCCATCGTCTTATGCGGATGGCGGGGAAAGGCCTCCATTCGAACTTTTGTGCCCAAGAATGAACGGCTTCATTATCTCAGGATGATGGGGCTGGAGGTATTGGGAGAAAAGAAGAAGGAAGGGGTTATCCTCACAAATGAGAGTGCAGCCAGCACCGGACAGCCAGACAATGATGTGACTGAGGGACAGAGAGCAGGAGAGCCCAACAGCCCAGATGCAGAAGAGGCCAACAGTCCAGACGTGACAGCAGGCTGTGACCCGGCGGGGGTCCATCCACCCCGGTGAAATTCCTGTGGAATGTGTGTCAGTTAGGGTGTGGAAAGTCCCCAGGCTCCCCAGCAGGCAGAAGTATGCAAAGCATGCATCTCAATTAGTCAGCAACCAGGTGTGGAAAGTCCCCAGGCTCCCCAGCAGGCAGAAGTATGCAAAGCATGCATCTCAATTAGTCAGCAACCATAGTCCCGCCCCTAACTCCGCCCATCCCGCCCCTAACTCCGCCCAGTTCCGCCCATTCTCCGCCCCATGGCTGACTAATTTTTTTTATTTATGCAGAGGCCGAGGCCGCCTCTGCCTCTGAGCTATTCCAGAAGTAGTGAGGAGGCTTTTTTGGAGGCCTAGGCTTT | | | | | |
