# Supplementary material for: The m5C methyltransferase NSUN2 promotes codon‐dependent oncogenic translation by stabilising tRNA in anaplastic thyroid cancer
Source: Clin Transl Med. 2023 Nov 20;13(11):e1466. doi: 10.1002/ctm2.1466 (PMC10659772; doi:10.1002/ctm2.1466)
Supplement: Supplementary file 6 — Supporting information [file CTM2-13-e1466-s005.docx]

| **Supplemental Table 4 Antibodies and chemicals** | | | | | |
| --- | --- | --- | --- | --- | --- |
| **REAGENT or RESOURCE** | **SOURCE** | **IDENTIFIER** | **Source** | **Calculated MW** | **Observed MW** |
| **Antibodies** | | | | |  |
| TRAF2 | Santa Cruz Biotechnology | Cat# sc-7346; RRID: AB_628389 | Mouse | 50 kDa | 55 kDa，43 kDa |
| Bcl-2 | Santa Cruz Biotechnology | Cat# sc-7382; RRID: AB_626736 | Mouse | 26 kDa |  |
| Jun B | Santa Cruz Biotechnology | Cat# sc-8051; RRID: AB_2130023 | Mouse | 39 kDa |  |
| p53 | Santa Cruz Biotechnology | Cat# sc-126; RRID: AB_628082 | Mouse | 53 kDa |  |
| c-MYC | Proteintech | Cat# 10828-1-AP; RRID: AB_2148585 | Rabbit | 49 kDa | 62-65 kDa, 50 kDa |
| RAB31 | Proteintech | Cat# 16182-1-AP; RRID: AB_1851568 | Rabbit | 22 kDa | 22 kDa |
| NSUN2 | Proteintech | Cat# 20854-1-AP; RRID: AB_10693629 | Rabbit | 86 kDa | 90-100 kDa |
| Vimentin | Servicebio | Cat# GB11192; RRID: AB_2814685 | Rabbit | 57 kDa | 57 kDa |
| Beta Tubulin | Servicebio | Cat# GB11017B; RRID: | Rabbit | 55 kDa | 55 kDa |
| N Cadherin | Servicebio | Cat# GB111009; RRID: | Rabbit | 100 kDa | 140 kDa |
| E Cadherin | Servicebio | Cat# GB11868; RRID: | Rabbit | 98 kDa | 124 kDa |
| GAPDH | Servicebio | Cat# GB11002; RRID: AB_2904017 | Rabbit | 37 kDa | 37 kDa |
| PIK3CA | ABclonal | Cat# A12484; RRID: AB_2759327 | Rabbit | 124 kDa | 110 kDa |
| Puromycin | MEMD Millipore corporation | Cat# MABE343; RRID: AB_2566826 |  |  |  |
| Rabbit Control IgG | ABclonal | Cat# AC005; RRID: AB_2771930 |  |  |  |
| HRP conjugated Goat Anti-Mouse IgG | Servicebio | Cat# GB23301; RRID: AB_2904020 |  |  |  |
| **Chemicals** | | | | | |
| Western Protein Marker I | Servicebio | Cat# G2086 |  |  |  |
| Phosphate Buffered Saline, Powder | biosharp | Cat# BL601A |  |  |  |
| SDS-PAGE Running Buffer powder | Servicebio | Cat# G2018 |  |  |  |
| Tris Buffered Saline (TBS) powder | Servicebio | Cat# G0001 |  |  |  |
| SDS-PAGE Transfer Buffer powder | Servicebio | Cat# G2017 |  |  |  |
| Xylene | Sinopharm Chemical Reagent Co., Ltd. | Cat# 10023418; CAS: 1330-20-7 | AR |  |  |
| Neutral balsam | Sinopharm Chemical Reagent Co., Ltd. | Cat# 10004160; CAS: 96949-21-2 | FMP |  |  |
| Isopropanol | Sinopharm Chemical Reagent Co., Ltd. | Cat# 80109218; CAS: 67-63-0 | AR |  |  |
| Ethanol absolute | Sinopharm Chemical Reagent Co., Ltd. | Cat# 10009218; CAS: 64-17-5 | AR |  |  |
| 1%hydrochloric acid alcohol | Auragene | Cat# P013E |  |  |  |
| Methanol | GHTECH | Cat# 1.17001.023; CAS: 67-56-1 | AR |  |  |
| Cisplatin | Selleckchem | Cas#: 15663-27-1 | AR |  |  |
| Doxorubicin HCl | Selleckchem | Cas#: 25316-40-9 | AR |  |  |
| Sodium periodate | MACKLIN | S817518-25g  Lot#: C14675024 CAS: 7790-28-5 | AR |  |  |
| RPMI-1640 medium | Gibco | Cat#: 11875093 |  |  |  |
| Penicillin-Streptomycin Solution (5,000 U/mL) | Gibco | Cat#: 15070063 |  |  |  |
| 0.25% Trypsin (EDTA+, Phenol red+) | Servicebio | Cat: G4001-100ML |  |  |  |
| **Critical commercial assays** | | | | | |
| Dual Luciferase Reporter Assay Kit | Vazyme | Cat# DL101-01 |  |  |  |
| Evo M-MLV RT Mix Kit with gDNA Clean for qPCR | Accurate Biology | Code No. AG11728 |  |  |  |
| SYBR Green Premix Pro Taq HS qPCR Kit （Rox Plus） | Accurate Biology | Code No. AG11718 |  |  |  |
| AG RNAex Pro Reagent | Accurate Biology | Code No. AG21101 |  |  |  |
| miRNA 1st strand cDNA synthesis kit | Accurate Biology | Code No. AG11717 |  |  |  |
| SYBR Green Premix Pro Taq HS qPCR Kit II （Rox Plus） | Accurate Biology | Code No. AG11719 |  |  |  |
| rtStar^TM^ tRNA-optimized First Strand cDNA Synthesis Kit | Arraystar | Cat#: AS-FS-004 |  |  |  |
| Yeast tRNA Phe | Sigma-Aldrich | Cat#: R4018 |  |  |  |
